# Potentially bioavailable iron produced through benthic cycling in glaciated Arctic fjords of Svalbard

**DOI:** 10.1038/s41467-021-21558-w

**Published:** 2021-03-01

**Authors:** Katja Laufer-Meiser, Alexander B. Michaud, Markus Maisch, James M. Byrne, Andreas Kappler, Molly O. Patterson, Hans Røy, Bo Barker Jørgensen

**Affiliations:** 1 Center for Geomicrobiology, Department of Biology, Aarhus University, Aarhus, Denmark; 2 Center for Applied Geosciences, University of Tübingen, Tübingen, Germany; 3 Department of Geological Sciences and Environmental Studies, Binghamton University, New York, USA; 4 Present Address: GEOMAR, Helmholtz Centre for Ocean Research Kiel, Kiel, Germany; 5 Present Address: Bigelow Laboratory for Ocean Sciences, Maine, USA; 6 Present Address: School of Earth Sciences, University of Bristol, Wills Memorial Building, Bristol, UK

**Keywords:** Element cycles, Element cycles, Geochemistry

## Abstract

The Arctic has the highest warming rates on Earth. Glaciated fjord ecosystems, which are hotspots of carbon cycling and burial, are extremely sensitive to this warming. Glaciers are important for the transport of iron from land to sea and supply this essential nutrient to phytoplankton in high-latitude marine ecosystems. However, up to 95% of the glacially-sourced iron settles to sediments close to the glacial source. Our data show that while 0.6–12% of the total glacially-sourced iron is potentially bioavailable, biogeochemical cycling in Arctic fjord sediments converts the glacially-derived iron into more labile phases, generating up to a 9-fold increase in the amount of potentially bioavailable iron. Arctic fjord sediments are thus an important source of potentially bioavailable iron. However, our data suggests that as glaciers retreat onto land the flux of iron to the sediment-water interface may be reduced. Glacial retreat therefore likely impacts iron cycling in coastal marine ecosystems.

## Abstract (web summary)

The impacts of a melting Arctic on the biogeochemistry of marine ecosystems are unknown. Here, the authors investigate glacial input of iron to Svalbard fjords finding that reworking of glacial iron in fjord sediment is important to make iron bioavailable, but could be susceptible to glacial retreat.

## Introduction

The Arctic regions are warming 2–3 times faster than the global average^1^. Arctic glaciated fjord ecosystems, which are hotspots of carbon cycling and burial^2,3^, are predicted to be extremely sensitive to this warming^4^. Iron is an essential nutrient for phytoplankton and limits primary productivity across 30–40% of the global ocean area^5^. The Arctic Ocean is generally not considered to be iron-limited^6^. However, in the proximal North Atlantic Ocean, as well as the Nansen Basin and eastern Fram Strait of the Arctic Ocean, recent research indicates that seasonal and/or regional iron-limitation could exist and be exacerbated with climate change^7–9^.

Glaciers and ice sheets are a primary iron source to the oceans, along with rivers, hydrothermal vents, and aeolian dust^10–13^. The majority of glacially derived iron is in the particulate form or will rapidly become particulate once in contact with oxic and saline fjord water due to oxidation and flocculation, resulting in up to 95% of glacially sourced iron settling to fjord sediments after entering the marine environment^14–16^. It was found that iron-delivery by glaciers is dependent on bedrock geology and that benthic iron cycling is active in these sediments, which could play a significant role in the production of bioavailable iron^17,18^. However, the amount and physical-chemical characteristics of glacial iron, as well as its fate once delivered to the fjords, still remain poorly constrained^10,19^. The physical-chemical characteristics that determine if iron minerals will be available for biological processes are related to the speciation, particle size, surface area, and crystallinity^20,21^. Neutral-buffered ascorbate selectively extracts poorly crystalline, highly labile Fe(III) and surface-bound Fe(II)^22^, which are potentially bioavailable for phytoplankton^15,23,24^ and favorable for microbial reduction^25,26^. Surface-bound Fe(II) has been found to be a minor fraction of iron in Svalbard fjord sediments^27^. Therefore, we assume that the most neutral-buffered ascorbate-extractable iron (FeA) is Fe(III). While previous studies have focused on the delivery of iron to marine ecosystems by icebergs^28,29^ and proglacial meltwater^13,30^, these sources contain low amounts of FeA (0.75-26 µmol per gram dry weight (g dw^−1^))^13,28–30^, compared to what has been reported for fjord sediments (9.5–176 µmol  g dw^−1^)^26,31^. It is not well understood how benthic processes in fjord sediments are impacted by the input of glacial iron and, conversely, how benthic processes impact the physical and chemical characteristics of glacially derived iron. Determining what happens after glacially derived iron settles to fjord sediments is crucial to evaluate if these sediments function as a net source or sink of potentially bioavailable iron.

Iron is not simply buried in marine sediments after deposition. An interplay of biotic and abiotic reactions drive the benthic biogeochemical iron cycle and transform the speciation, mineralogy, and physical-chemical characteristics of iron^32,33^. The reduction of Fe(III) in sediments is catalyzed by abiotic redox reactions with sulfide or organic matter, and by biotic redox reactions mediated by microorganisms^33^. Microorganisms preferentially reduce labile, poorly crystalline Fe(III) minerals during organic carbon mineralization due to the high energy yield and relatively large surface area. However, over longer timescales microorganisms can also reduce highly crystalline Fe(III) minerals, such as hematite or iron in silicates^25,34^. Fe(II) is oxidized by abiotic reactions with oxygen, Mn(IV)-oxides or reactive nitrogen species, and by biotic reactions mediated by microorganisms. Microorganisms are thought to preferably oxidize dissolved Fe(II) with oxygen or nitrate as electron acceptors or coupled to anoxygenic photosynthesis^33^, while producing highly labile, poorly crystalline, biogenic Fe(III) minerals^35^. However, solid-phase Fe(II) in silicate or sulfide minerals is also available for oxidation by microorganisms^36^, and abundant in glacial systems^20,37^. The benthic iron cycle is connected to many other element cycles^33^. Thus, changes to the iron cycle create a cascade of impacts for example on the availability of phosphate and other nutrients and, most importantly, on the cycling and burial of carbon.

With ongoing warming, glacier termini are retreating from the sea onto land, which will lead to changes in the processing and export of glacially derived material^38^. The glacial retreat also causes changes in water circulation and primary productivity in the fjord ecosystem^39–41^. Arctic fjords are significant sinks of carbon^2,3^. It remains unknown how glacial retreat onto land will impact the processing of glacially sourced iron in fjord sediments and affect its speciation, transport and bioavailability. If iron cycling in fjord sediments is sensitive to glacial retreat, then this could change iron supply to fjord waters, and thereby have profound effects on fjord primary productivity and carbon cycling.

Here we show the effects of glacially derived iron on benthic processes in fjord sediments. Specifically, we quantify how benthic processes change the characteristics of glacially derived iron, and discuss the potential impacts of benthic processes on iron bioavailability and export to the water column. We quantified the amount of potentially bioavailable, ascorbate-extractable iron (FeA) and microbially reducible iron (FeM)^26,42,43^ using time-course extractions. We chose the time-course extractions because the dissolution kinetics of the iron during the extractions provide valuable data on the amount (M_(0)_), reducibility (v/a), lability (initial rate, taking into account the amount and reducibility), and composition (1 + 1/v, impacted by the mineralogy, particle size and crystallinity, where a value close to 1 shows the uniform composition and a value >1 shows a more complex composition) of the extracted iron pool (Table 1). While classically all FeA is defined as labile, with the lability parameter we can quantify how labile the FeA pool is, which allows for a more detailed comparison. Moreover, we used sequential end-point HCl extractions, which preserve the redox speciation of the extracted Fe, to measure the poorly crystalline (0.5 M HCl) and crystalline (6 M HCl) sedimentary iron pool, together yielding the total HCl-extractable iron (Table 1). Finally, we used ^57^Fe Mössbauer spectroscopy for the identification of iron-mineralogy within the glacial source and sediment samples. We find that the amount and reducibility of FeA and FeM from glacial sources (icebergs, proglacial rivers, proglacial plumes) is relatively low, while the amount and reducibility of FeA and FeM in the fjord sediments increases with distance from the fjord head. This pattern of increasing FeA and FeM holds across three Svalbard fjords with differing glacial regimes and catchment geology. We conclude that fjord sediments are a bioreactor for authigenic labile iron production and that this process is enhanced from the fjord head to mouth. Moreover, fjord sediments represent a potentially important, but yet overlooked, source of potentially bioavailable iron to the overlying water column; however, the iron cycle is sensitive to glacial retreat which may reduce the fjord sediments role in supplying potentially bioavailable iron to the water column.

**Table 1:** Description of the abbreviations and parameters of the applied Fe-extrication methods.

Extraction method	Pool of iron extracted	Parameters of the extraction method	Meaning of the parameter (abbreviation)
Ascorbate Fe reduction time-course extractions	Ascorbate extractable iron (FeA)	M_(0)_	Amount of FeA
		1 + 1/v	Composition FeA
		v/a	Reducibility of FeA
		Initial rate	Lability of ascorbate extractable iron
Microbial Fe reduction time-course extractions	Microbially extractable iron (FeM)	M_(0)_	Amount of microbially extractable iron
		1 + 1/v	Composition of microbially extractable iron
		v/a	Reducibility of microbially extractable iron
		Initial rate	Lability of microbially extractable iron
Sequential HCl extractions	HCl-extractable iron (FeHCl)	0.5 M HCl Fe(II)	
		0.5 M HCl Fe(III)	
		0.5 M HCl Fe(total)	
		6 M HCl Fe(II)	
		6 M HCl Fe(III)	
		6 M HCl Fe(total)	
		Total FeHCl	

## Results and discussion

### Composition of iron in glacial sources

**Fig. 1:**
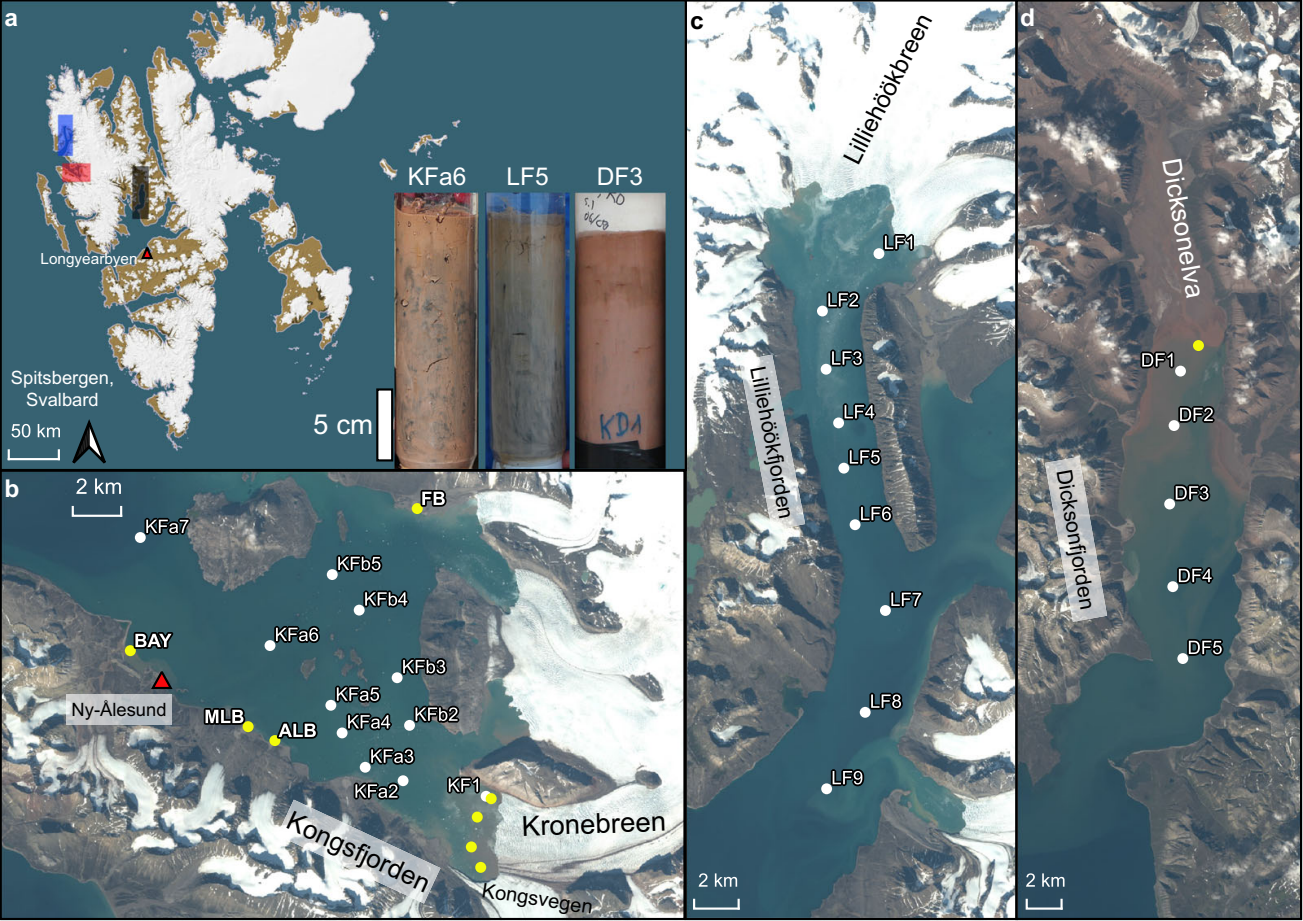
Map of sampling stations. **a** Overview map of Svalbard, with the three investigated fjords indicated by colored rectangles, and examples of a sediment core from each fjord (Kongsfjorden = red, core KFa6; Lilliehöökfjorden = blue, core LF5; Dicksonfjorden = gray, core DF3). **b**–**d** Maps of sampling stations in the individual fjords; **b** Kongsfjorden, **c** Lilliehöökfjorden, **d** Dicksonfjorden. White dots represent sediment sampling stations. Yellow dots represent glacial source sampling stations; BAY Bayelva river, MLB Midre Lovenbreen, ALB Austre Lovenbreen, FB Ferringbreen. The yellow dots without labels indicate plume and iceberg samples. Red triangles show the location of NyAlesund and Longyearbyen for orientation. 10-m satellite imagery from Sentinel-2 taken on 2 August 2017.

**Fig. 2:**
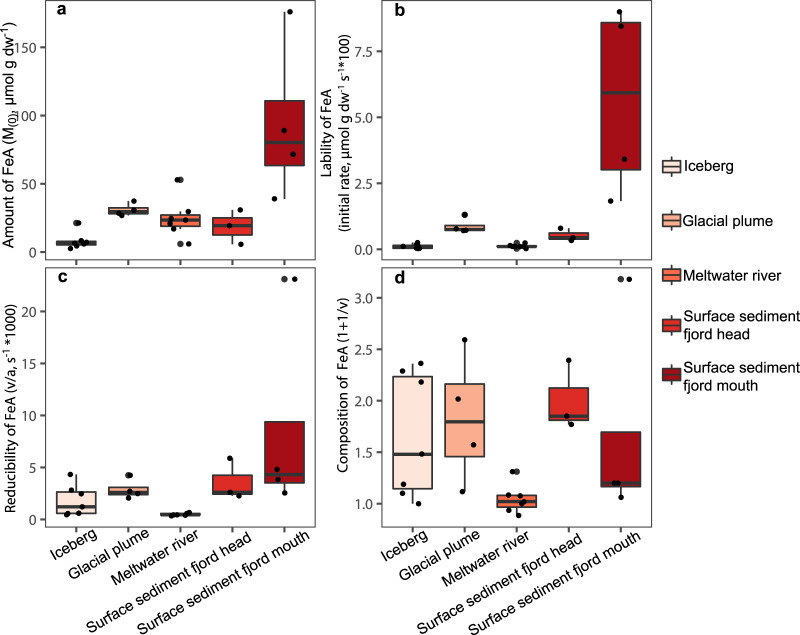
Amount and characteristics of ascorbate-extractable Fe (FeA) from the glacial source material and fjord surface sediment. All data points are shown as black dots. The red boxes show the interquartile range and the thick horizontal line in the boxes shows the median. The vertical line at the top and the bottom of the boxes shows the maximum and the minimum, defined as 1.5× interquartile range. **a** Amount of FeA, **b** lability of FeA, **c** reducibility of FeA, and **d** composition of FeA. Surface sediment values are from 0 to 1 cm sediment depth.

Particulate material collected from a variety of glacial sources in Kongsfjorden had a high total iron content (320–1400 µmol total HCl extractable Fe g dw^−1^, Supplementary Table 1, Fig. 1), which is within the range previously reported for glacial sources worldwide (500–1600 µmol g dw^−1^)^11^. However, iron minerals in these glacial sources contained relatively low amounts of FeA (Table 1), with 30.9 ± 4.6, 28.1 ± 12.9, and 8.1 ± 6.1 µmol g dw^−1^ in particulate material from the proglacial plumes, meltwater rivers, and iceberg samples, respectively^26^ (Fig. 2a). Fe mineralogical composition detected by ^57^Fe Mössbauer spectroscopy also showed that a large proportion of iron in the glacial source material is highly crystalline and not extractable by ascorbate. Iron in a Kongsfjorden plume sample had a relative abundance of 17.8 ± 1.6% hematite, whereas material from a Kongsfjorden iceberg contained about twice as much hematite, accounting for 41.3 ± 1.9% of the iron pool (Supplementary Fig. 1, Supplementary Table 2). These data corroborate results of Raiswell and coworkers^15^, who showed that labile iron produced by chemical and biological weathering in subglacial systems gets slowly converted into less labile phases such as goethite or hematite in glacial ice^15^. Alternatively, this could be a function of the hematite content of the Devonian-age red sandstone underlying the glaciers at the head of Kongsfjorden^44^, which is being crushed and directly incorporated into glacial ice. Glacial sources contain relatively low amounts of FeA, such that only a small fraction (0.6–12%, average 3.3%) of glacially derived iron is potentially bioavailable for phytoplankton^23^. These data are in agreement with previous results showing a high total iron content, but a low amount of FeA in glacial sources of Kongsfjorden^15,29^. The amount of FeM was about two times higher than FeA (Supplementary Table 1), consistent with what has been previously found^26^ and attributed to the large surface area of these glacial sediments being favorable for microbial reduction, differences in the mineral phases available to biotic and abiotic processes, and differences in mechanisms of biotic and abiotic iron reduction^26^. Still, FeM, representing the fraction of iron that is available for a microbial reduction under ideal, non-limiting laboratory conditions^26^, was a small fraction of the total iron in these samples (1.1–30%, average 9.4%).

Samples from the same type of glacial source in Kongsfjorden generally contained a similar amount of FeA, but there were some exceptions (Fig. 2a). The iceberg and meltwater plume samples from Kongsvegen contained a higher (3.6 and 1.3-fold, respectively) amount of FeA compared to the samples from Kronebreen (Supplementary Table 1). The differences in FeA content found in Kongsvegen and Kronebreen samples are likely to be caused by differences in bedrock geology. Kongsvegen overrides Carboniferous-Permian age limestones and dolostones, whereas Kronebreen overrides iron-rich, Devonian age red sandstones^44^, resulting in strikingly different colors of the sediment (Supplementary Fig. 2). It is also possible that these differences could be a function of differential processing of material within the two glacial systems before delivery to the fjord. Samples from Austre Lovenbreen, which were collected in consecutive years, also contained variable amounts of FeA (Supplementary Table 1). However, the parameters determined in FeA extractions (lability, reducibility, and composition) were similar. This indicates that the pool of FeA from the Austre Lovenbreen catchment in both years was similar, but more dilute in one year compared to the other. In general, we found that samples from the same type of glacial source contained FeA with similar characteristics, as can also be seen from the similar shape of the dissolution curves (Supplementary Fig. 3).

The composition of FeA describes the diversity (1 + 1/v > 1) or uniformity (1 + 1/v close to 1) of the mixture of Fe minerals contained within the sediment in terms of mineralogy, particle size, and/or crystallinity. The composition of FeA was highest for the particles from the proglacial plume at the head of Kongsfjorden and the lowest for the meltwater rivers in Kongsfjorden (1 + 1/v of 1.83 ± 0.6 and of 0.99 ± 0.1, respectively, Fig. 2d). These results indicate that glacial iron transported by proglacial rivers gets sorted or chemically or physically modified, such that a uniform type of iron mineral is supplied by these rivers, whereas proglacial plumes emanating from beneath the marine-terminating glacier deliver a more diverse pool of FeA. Kongsfjorden glacial plume contained the highest FeA lability of glacial source material, which was 8-fold higher compared to the average of the icebergs (8.8 × 10^−3^ ± 2.9 × 10^−3^ µmol g dw^−1^ s^−1^, Fig. 2b). This highlights that glacial meltwater emanating in front of Kronebreen, contains FeA that was produced by subglacial processes that promote the production of potentially bioavailable Fe, such as biogeochemical weathering^15^. Despite this, the Kongsfjorden glacial source sample with the highest lability of FeA is ten times lower than the highest reported values of Kongsfjorden sediment^26^ (Fig. 2).

Particulates collected in the plume of the meltwater river at the head of Dicksonfjorden (Dicksonelva, Fig. 1) had only a fifth of the amount of FeA that was found in the plumes of Kongsfjorden meltwater rivers. The amount of FeA in Dicksonelva particulates (5.95 µmol g dw^−1^, Fig. 2a) is similar to the average of the Kongsfjorden iceberg samples. Also, the lability and reducibility of FeA of Dicksonelva particulates were most similar to the lowest values that we found for the icebergs in Kongsfjorden. The lability of FeA in Dicksonelva particulates was only one fourth of what we measured for Kongsfjorden river particulates and 25-times lower compared to the proglacial plume in Kongsfjorden. The composition of FeA (1 + 1/v of 1.31) was more complex than the meltwater rivers in Kongsfjorden (Supplementary Table 1). Dicksonelva is different from the meltwater rivers in Kongsfjorden as it enters the fjord in a large delta with an intertidal mudflat^45^, which seems to affect the transport and/or transformation of FeA. Previous studies have concluded that sediment transport in meltwater rivers will transform minerals into more labile phases due to increased weathering^27^. We hypothesize that, as the amount and lability of FeA were most similar to iceberg samples from Kongsfjorden, and much lower than in all other measured meltwater rivers, this does not seem to hold true for Dicksonelva.

Our results show that only a limited fraction of the total iron delivered by glaciers is potentially bioavailable iron, independent of the glacial regime or source type. We also find that the characteristics (reducibility, lability, and composition) of iron delivered by different glacier and glacial source types, differ. Based on these findings we conclude that glacial retreat and shrinkage could lead to changes in the amount and characteristics of iron that is delivered to fjords.

### Spatial distribution of Fe in Kongsfjorden sediment

**Fig. 3:**
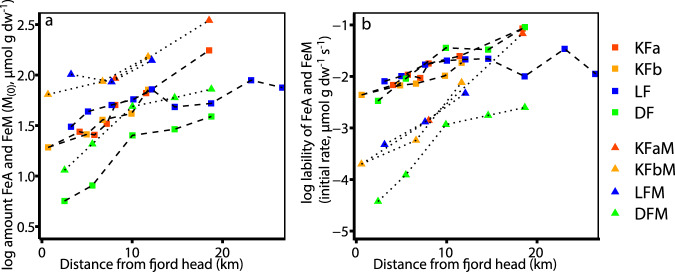
Amount (M_(0)_) and lability (initial rate) of ascorbate extractable Fe (FeA) and microbially extractable Fe (FeM) versus distance from the fjord head. **a**, **b** Amount (M_(0)_) and reducibility (initial rate), respectively, of FeA (squares) and FeM (triangles) in surface sediment.

The amount and lability of FeA at the fjord head (KF1; Fig. 1) were the lowest of all surface sediment samples within the Kongsfjorden transects (Fig. 3). FeA at KF1 was similar to the mean of Kongsfjorden glacial sources (Figs. 2 and 3) and implies there is little processing of iron at the head of the fjord, likely because the sedimentation rates are very high close to glacial meltwater inputs^14^. The amount and lability of FeA in surface sediment in Kongsfjorden increased by ninefold and 19-fold, respectively, at the station furthest away from the fjord head compared to the station closest to the fjord head in the southern (KFa) transect (Fig. 3). A similar increase in FeA amount and lability over distance was found in the northern (KFb) transect of Kongsfjorden (Fig. 3). These increases are exponential as seen from the linear increase in the semi-log-plot (Fig. 3) and an *R*^2^ of 0.96 and 0.94 for the KFa and KFb transects, respectively, when fitting an exponential model through the data (Supplementary Table 3). FeM showed even more pronounced differences in the amount and lability at the surface of station KFa7 and KFb5 compared to all the sources (Fig. 3). These increases in FeA and FeM are either produced by preferential transport of the smallest and most labile particles, a reworking of particles during transport in the water column, or by the processing of the iron upon sedimentation, producing authigenic FeA and FeM.

The composition of FeA in the transects became more complex over the first few km distance (from a 1 + 1/v of 1.85 at KF1 to 2.18 and 2.06 at KFa4 and KFb3, respectively), likely due to the glacial sources containing FeA with varying composition and authigenic production of FeA within the sediment. At stations with a greater distance to the fjord head, the composition of FeA became more homogeneous again, reaching values as low as 1.20 at KFa7 and KFb5, indicating the presence of a more uniform pool of highly labile iron (Fig. 2). These changes imply that significant processing of iron occurred after sedimentation at stations further away from the fjord head, likely through microbial dissimilatory iron reduction or interactions with sulfide^27,31^. Our data show that a homogenous pool of highly labile and potentially bioavailable FeA is accumulating in the surface sediments of Kongsfjorden as the distance from the glacial source increases (Fig. 2 and S4).

These results are consistent with ^57^Fe Mössbauer spectroscopy, which showed that the content of labile and crystalline iron minerals in KF1 sediment was similar to the Kronebreen plume (e.g., 21.1 ± 1.3 and 17.8 ± 1.6% of hematite was found in KF1 sediment and the Krobebreen plume, respectively). The mineral composition of KFa7 sediment was different from KF1 and the Kronebreen plume, with lower proportions of crystalline minerals (e.g., only 10.3 ± 2.1% hematite, Supplementary Fig. 1, Supplementary Table 2). This distribution of iron minerals with different crystallinities could be caused by the transport of the finest and most labile particles to the more distant stations, which would also explain the higher lability of FeA. However, the lability of FeA in KFa7 surface sediment is higher than any value measured in the proglacial plume, and also notably higher (8.6-fold) than the average of all glacial sources (Fig. 2b). Taken together, these data indicate that the abundant iron mineral species were dominated by less crystalline, more labile iron phases further from the head of the fjord and that they might be authigenic.

### Catchment geology impact on Fe cycling

An increase in the amount of FeA over distance from the fjord head was also found in Lilliehöökfjorden, reaching a maximum of 89 µmol g dw^−1^ at LF8, which is a 3.9-fold increase within the 23 km transect (Fig. 3). The lability of FeA also increased 4.3-fold in our Lilliehöökfjorden transect (Fig. 3). FeM shows the same increasing trend in amount and reducibility (Fig. 3). The catchment geology of Lilliehöökfjorden is dominated by metamorphic rocks such as marble, mica-schist, and minor amounts of quartzite of Middle Proterozoic age, while Kongsfjorden is dominated by Devonian red sandstone as well as Carboniferous-Permian age dolostone and limestone^44^. Still, the same patterns of an increasing amount of lability of FeA were found in two fjords, despite the diversity of bedrock types supplying material to each fjord. This pattern of increasing labile iron content with distance from the fjord head was also observed in two fjords in southwestern Svalbard (determined by hydroxylamine-hydrochloride extractions)^27^. Van Mijenfjorden and Van Kuelenfjorden in southern Spitsbergen, Svalbard drain even more diverse bedrock assemblages and reinforce the widespread nature of these patterns in fjord sediments. The increases we observe in Kongsfjorden are statistically significant over the entire length of the transects (Supplementary Table 3). However, the increase of FeA within Lilliehöökfjorden is interrupted where Möllerfjorden and Lilliehöökfjorden merge (between LF6 and LF7; Figs. 1 and 3), likely due to nearby glaciers from Möllerfjorden supplying less labile iron to the sediments. The increases in the amount and lability of FeA are significant if only the data until LF5 are included in the analysis. If we consider the entire transect, including stations from LF6 and outward, which are impacted by Möllerfjorden, the amount of FeA over distance has low significance and the increase in the lability over distance has no significance. (Supplementary Table 3). These data show the sensitivity of fjord sediments to nearby glacial meltwater inputs.

The composition of the Lilliehöökfjorden FeA pool develops in a manner similar to the two transects in Kongsfjorden, where a diverse pool of FeA becomes progressively more uniform in composition with distance from the fjord head (Supplementary Fig. 4). No hematite could be identified by ^57^Fe Mössbauer spectroscopy in Lilliehöökfjorden samples and the iron mineral composition was different from Kongsfjorden as expected from the different bedrock assemblage and sediment color (Fig. 1, Supplementary Fig. 5, and Supplementary Table 2). Collected spectra were similar for LF1 and LF5, with a higher proportion of Fe(III) towards the fjord mouth (LF5) compared to the fjord head (LF1). This increase in the relative abundance of Fe(III) detected by ^57^Fe Mössbauer spectroscopy supports the trend of increasing authigenic Fe(III) minerals in sediment nearer the fjord mouth as also determined with Fe extractions. Consequently, the oxidation of glacially derived Fe(II) to Fe(III), by biotic or abiotic processes^33^, appears to be important for the production of FeA in Lilliehöökfjorden. In conclusion, the increases of FeA and FeM in fjord sediments toward the fjord mouth, irrespective of catchment geology, reveals a gradual transformation of glacially derived Fe into more labile phases. We predict this to be a widespread process in glaciated fjords.

### FeA and FeM production through benthic cycling

Sedimentation gradients in Arctic fjords caused by particle transport in freshwater lenses provide one possible explanation for the observed gradients in FeA and FeM by carrying the finest and most labile particles furthest^14^. However, we did not find evidence for long-distance transport of the finest and most labile glacial iron in the observed gradients. Over 95% of the grain size distributions from surface sediment samples recovered along the transects are characterized by silt and clay (<63 µm or 4 ϕ) (Supplementary Fig. 6). We found no systematic relationship between the percent of fine-grained material and the distance from the fjord head (Supplementary Fig. 6, further information see SI). There is a small decrease in grain size over the first 8 km of the transect, but there is no change after that, while FeA and FeM still continue to increase beyond this point (Fig. 3). Fe cycling within Fe-organic matter aggregates suspended in the water column could increase iron lability during transport. However, we argue that the time to deposition is relatively short in these shallow fjords (<350 m), compared to the deep, open ocean where Fe-organic matter aggregate studies have been previously conducted^46,47^. Compared to the time available for cycling in the sediments afterwards, the impact of cycling in the water column is likely minor. We conclude that the increase of FeA and FeM over distance cannot be explained as a function of the preferential transport of small and reactive particles or transformation during transport through the water column.

**Fig. 4:**
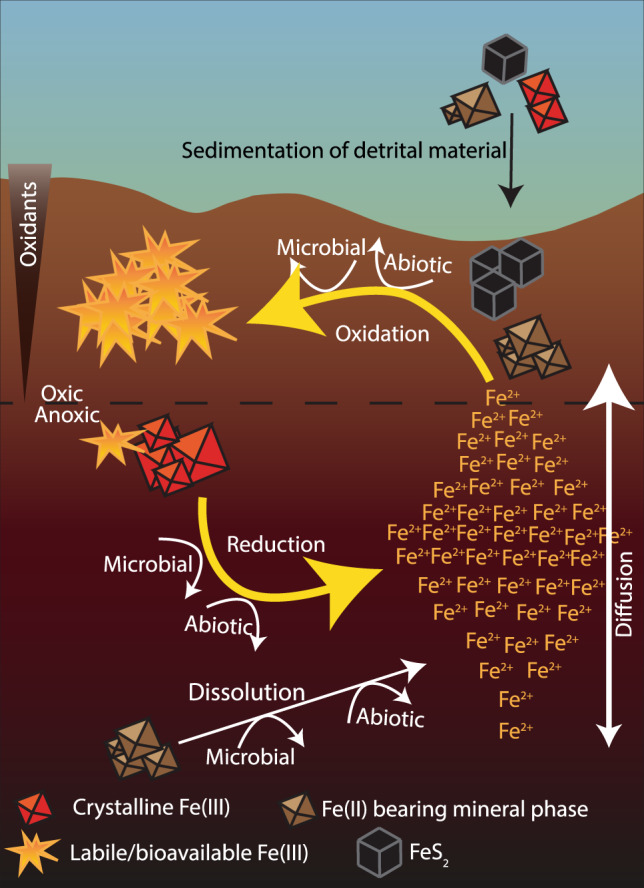
Schematic figure of benthic iron cycling. Illustration of the production of ascorbate-extractable Fe (FeA) and microbially extractable Fe (FeM), which is labile and potentially bioavailable Fe, at the oxic-anoxic interface in fjord sediments. These sediments are unique to other marine sediments, as glacial input provides huge quantities of Fe(III) and Fe(II) bearing minerals to the sediment surface.

**Fig. 5:**
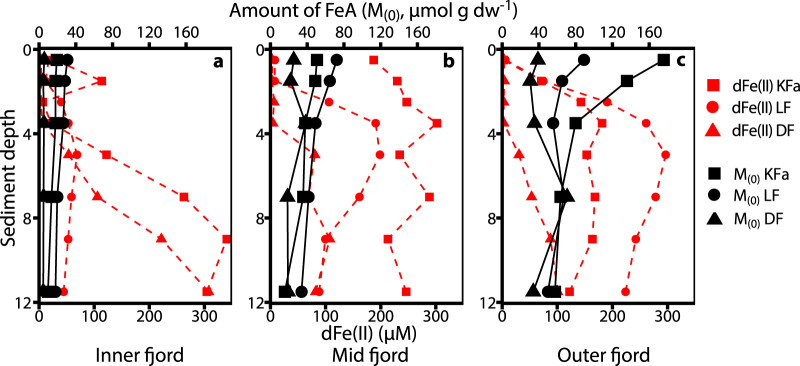
Concentrations of ascorbate-extractable Fe (FeA) and dissolved iron over sediment depth at three stations within the transects. The three frames show the amount of FeA and the concentration of dissolved iron (dFe(II)) in the pore water versus sediment depth at **a** stations closest to the fjord head (Kongsfjorden (KFa) = KFa1, Lilliehöökfjorden (LF) = LF1, Dicksonfjorden (DF) = DF1); **b** mid-fjord stations (KFa = KFa5, LF = LF5, DF = DF3); and **c**: stations closest to the fjord mouth (KFa = KFa7, LF = LF8, DF = DF5).

We propose that the main driving force for the higher FeA and FeM content and lability of fjord sediments is benthic cycling through an interplay of abiotic and biotic processes^33^ (Fig. 4). These processes produce authigenic, poorly crystalline, highly labile, and easily reducible iron at the oxic sediment surface (Fig. 5), underlying oxic bottom waters, through abiotic or microbially mediated oxidation of dFe(II)^33^. Steep concentration gradients with sediment depth, driving a flux of dFe(II) into the oxic sediment layers, were found at all stations in Kongsfjorden and Lilliehöökfjorden (Supplementary Fig. 7). The source of Fe(II) is a combination of: (i) reductive dissolution of Fe(III) from Fe(III) (oxyhydr)oxides, (ii) pyrite, originating from the bedrock beneath the glacier^18^, and (iii) dissolution of other Fe(II)-bearing minerals such as Fe(II)-carbonates or primary silicates through microbial or abiotic weathering processes^48^ (Fig. 4). Over timescales of years to decades these processes are likely to access the fractions of the glacially derived Fe, which initially are not extractable by ascorbate or microorganisms. Which of the possible Fe(II) sources is most important in the different fjord sediments is a function of the catchment geology, geochemical conditions, and microbial activity. Regardless of the source of Fe(II), we propose that Fe(II)-oxidation at the sediment surface produces authigenic Fe(III) minerals.

**Fig. 6:**
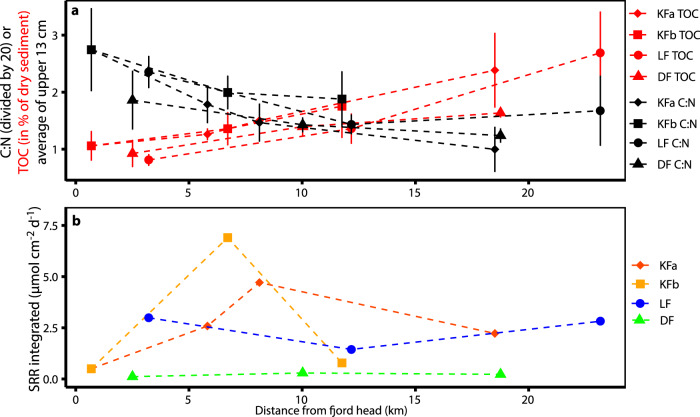
Total organic carbon (TOC), carbon to nitrogen (C:N) ratios, and integrated sulfate reduction rates (SRR) over distance from the fjord head. **a** Average of C:N values and TOC in the upper 13 cm of the sediment over distance from the fjord head. Error bars show SD. **b** Depth-integrated SRR over distance from the fjord head.

We observe a strong gradient in FeA and FeM from fjord head to mouth, indicating that the process that produces FeA and FeM at the sediment surface gets stronger and/or decreasing sedimentation rates provide more time for these processes with increasing distance from the glacial source. In glaciated fjords, steep gradients in hydrology, biology, and geochemistry exist due to inputs of glacial material at the fjord head and the marine influence at the fjord mouth^2,49^. At the fjord head, high sedimentation rates of detrital material and low primary productivity within a thin photic zone^14,50^, lead to sediment with low TOC content, caused by low primary productivity and the dilution effect of lithogenic material. We found organic carbon with a high C:N ratio of up to 70 in sediment close to the glacial source in Kongsfjorden (Fig. 6), originating from the terrestrial input of petrogenic organic carbon^51^. Petrogenic organic carbon is more resistant to microbial degradation compared to fresh marine organic carbon^52^. Terrestrial organic carbon usually has C:N values >20, while fresh marine organic matter usually has lower C:N values around 6–9^3^, and is more readily available to microbial degradation compared to terrestrial material^52^. Towards the fjord mouth TOC contents gradually increased, while C:N ratios decreased and approached a more marine-like signature (Fig. 6). Towards the mouth there is higher primary productivity and greater input from Atlantic water, leading to more marine organic matter settling to the sediment^50,51^. Similar trends of increasing TOC and decreasing C:N with distance from the fjord head were reported previously for Svalbard fjords^2,27^ and C:N values of up to 50 have also been reported for Greenlandic fjords^53^. We conclude that sediments close to glaciers can sustain only moderate activity of microbial Fe(III)- or sulfate-reduction due to the low amount and refractory characteristics of the organic carbon, which is supported by the low sulfate reduction rates (Fig. 6). Further from the glacier, the sedimentation rate of inorganic detrital material decreases and organic carbon export to the sediments increases, producing sediment with a higher TOC content and lower C:N^49^. The higher amount of organic carbon with a lower C:N ratio, as well as lower sedimentation rates, altogether create more favorable conditions for Fe-cycling at the sediment surface further away from the glacial source, as the organic carbon can support higher rates of microbial Fe(III) reduction and sulfate reduction (Fig. 6)^31^, both leading to the production of dFe(II).

**Fig. 7:**
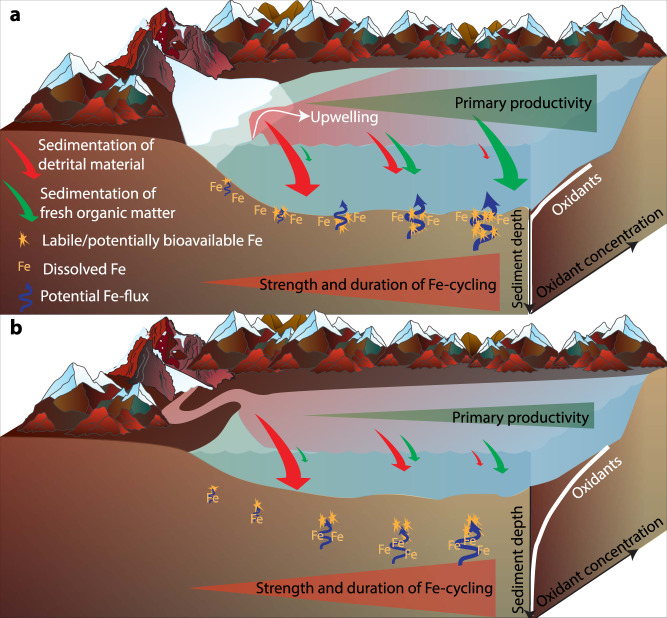
Schematic comparison of current and future scenarios. The strength of the processes is indicated by symbol/arrow size. **a** Fe cycling in a fjord with a marine-terminating glacier (current scenario) and **b** with a land-terminating glacier (future scenario). Fe-cycling is impacted by the gradients of input of detrital material and fresh organic matter. Symbol descriptions in panel a also apply to (**b**).

Based on our TOC and C:N results, we expected SRR to increase concurrently with the increase in TOC and the decrease in C:N. However, depth-integrated rates of SRR show no consistent increase with distance from the fjord head (Fig. 6). SRR do not increase as expected along with the TOC and C:N gradients, which is likely caused by the concurrent increase in FeA and FeM along the transect, enabling Fe-reducers to compete favorably with sulfate-reducers (for a more detailed discussion of SRR in relation to TOC and C:N, see SI). In addition to the increased activity of benthic iron cycling, the lower sedimentation rates in the outer part of fjords^49^ lead to a more abundant and active benthic fauna^54^, which further intensifies benthic cycling^55^. Lower sedimentation rates also lead to increased time for iron to be repeatedly cycled before it gets buried deeper in the sediment (Fig. 7a).

The increase in Fe lability through benthic cycling, also called “rejuvenation”, has been shown to be an important pathway for bioavailable iron production in continental margin sediments^55^. A similar pattern of increasing labile iron content was found in a transect from the shelf towards the deepest parts of the Guaymas Basin in the central Gulf of California^56^. The mechanism for this pattern was ascribed to various possible processes and partially to benthic cycling by microorganisms; however, there were numerous Fe sources, making it difficult to pinpoint a dominant process^56^. The unique situation of a clearly defined major source of iron (the glaciers) makes glaciated fjords ideal for investigating benthic iron cycling processes. We propose that the increasing intensity of benthic iron cycling is due to an increased amount of labile organic carbon and time before burial, which together contributes to the increases of FeA and FeM from fjord head to mouth. The steep gradients of FeA and FeM are produced by recycling of glacial Fe within the sediment and not derived directly from glacial sources. These gradients of FeA and FeM show that proximity to the marine-terminating glacier front is significant for iron cycling and the production of potentially bioavailable iron, making these processes sensitive to future glacial retreat.

### The impact of glacial retreat on FeA and FeM distribution

The general pattern of increasing amount and lability of FeA and FeM with distance from the fjord head was also observed in Dicksonfjorden, which is fed by land-terminating glaciers only (Fig. 1). The amount of FeA was only about half of what was found in Kongsfjorden and Lilliehöökfjorden at similar distances from the fjord head (Fig. 3a), probably because of the lower amount of FeA present in particulates in the plume at the head of Dicksonfjorden. However, the lability of FeA was within the same range as for Kongsfjorden and Lilliehöökfjorden (Fig. 3b), despite the lability in the plume at the head of Dicksonfjorden being much lower compared to the average of the glacial source samples from Kongsfjorden (Supplementary Table 1). We propose that, similar to Kongsfjorden and Lillihöökfjorden, benthic cycling is mainly responsible for the increase in the amount and lability of FeA from head to mouth in Dicksonfjorden. In contrast to Lilliehöökfjorden and Kongsfjorden, where the amount of FeA peaked at the sediment surface, the maximum concentration of FeA was never found at the sediment surface in Dicksonfjorden (Fig. 5). At station DF1, the amount of FeA did not change significantly over sediment depth, and at station DF3 and DF5 the maximum concentration of FeA was found at 3–4 and 6–8 cm sediment depth with 38.3 and 70.7 µmol g dw^−1^, respectively (Fig. 5). We conclude that the production of FeA is not only independent of bedrock lithology but also of the glacial regime. The specific depth-distribution of FeA and FeM that we found in Dicksonfjorden likely attenuates the recycling of potentially bioavailable iron to the water column.

The subsurface peaks of FeA in Dicksonfjorden are likely caused by deeper penetration of oxidants (such as oxygen, nitrate, or Mn(IV)-oxides; Supplementary Table 4), leading to the oxidation of dFe(II) producing authigenic FeA. This is further evidence that FeA is mainly authigenic and not a function of the fine-grained and labile material getting transported furthest or produced by cycling during transport in the water column. The presence of oxidants is evident from the absence of dissimilatory sulfate reduction, dFe(II), and dMn just above the depth where the maximum amount of FeA was found at station DF3 and DF5 (Fig. 5, Supplementary Fig. 8). This indicates that dFe(II) and dMn were oxidized within the top 3–5 cm of the sediment and could not reach the sediment surface at these stations. At station DF1, low SRR (<1 nmol cm^−3^ d^−1^) and dMn were found within the upper 4 cm of the sediment, but no dFe(II) was detected (Supplementary Fig. 8). Again, Dicksonfjorden is in contrast to Kongsfjorden and Lilliehöökfjorden where sulfate reduction was active and dFe(II) and/or dMn, could be detected within the upper 2 cm of the sediment at all stations (Figs. 5 and S8). The deeper penetration of oxidants in Dicksonfjorden sediments is likely caused by the generally lower primary productivity in fjords with land-terminating glaciers as they lack glacial upwelling, which is known to entrain nutrient-rich bottom water and transport it up to the photic zone where it supports primary productivity^39–41^. The diminished primary productivity in the Dicksonfjorden water column in the whole fjord leads to a smaller increase in TOC content of the sediment with distance from the head of the fjord compared to Kongsfjorden and Lilliehöökfjorden (Fig. 6a). The lower TOC content at stations further away from the fjord head also led to depth-integrated SRR that remained low over the entire transect (Fig. 6b). As a consequence of low TOC content, the sediment microbial community is less active and oxidants penetrate deeper into the sediment. This prevents dFe(II) from reaching the sediment surface to fuel authigenic labile Fe(III) production or diffuse into the water column. The observations from Dicksonfjorden suggest that glacial retreat onto land may have the potential to dampen the function of fjord sediments as a source of potentially bioavailable iron, but these results must be confirmed in other fjords with land-terminating glaciers.

### The effect of glacial retreat on Fe-export to the water column

Sediments are a dominant source of Fe for waters around coastal Greenland and Svalbard^9^. The production of authigenic, labile Fe(III) at the sediment-water interface^57–59^, and diffusion of dFe(II) across the sediment-water interface^60^, are important factors for Fe-transfer into the water column. Moreover, bioturbating and bioirrigating benthic fauna are abundant in the outer parts of the fjord^54^ and also contribute to Fe-transfer to the water column. Svalbard fjords are relatively shallow and none of the studied fjords has a pronounced sill near the mouth. Thus, the sediments are exposed to inflowing and/or outflowing seawater^49^, this physical contact of the sediment and the water is a prerequisite for the transfer of potentially bioavailable iron to the water column and the further transport of sediment-derived Fe onto the shelf and open ocean or back into the fjord. Within the fjord, subglacial discharge from marine-terminating glaciers drives the upwelling of Fe- and macronutrient-rich water, which are essential for phytoplankton growth in glaciated fjords^39–41,61^. The iron enrichment in the upwelled water could at least partially also result from fluxes of Fe from sediments into the fjord bottom water. We hypothesize that Fe released from sediments into the fjord bottom water is recycled into the photic zone by glacial upwelling. We furthermore hypothesize that there is a decreased potential for Fe-recycling into the photic zone in Dicksonfjorden compared to Kongsfjorden or Lilliehöökfjorden because (i) authigenic FeA is produced at several cm sediment depth, (ii) dFe(II) does not reach the sediment surface and (iii) glacial upwelling is lacking (Fig. 7). This hypothesis highlights the potential changes to fjord Fe cycling as a result of glacial retreat but needs further investigation and studies coupling benthic and pelagic processes are required to confirm this hypothesis. The hypothesized changes to the fjord Fe cycle can further disrupt the carbon cycle, as Fe is an essential nutrient for phytoplankton in the water column^5^ and Fe(III) is a significant anaerobic terminal electron acceptor for carbon remineralization in fjord sediments^33^. Therefore, glacial retreat may impact the biological carbon pump in fjords, due to the decreased potential of fjord sediments to function as Fe-sources to the water column and the lack of glacial upwelling.

### Fjord sediments are an active interface between land and ocean

To improve our understanding of iron cycling in the ocean and the production of bioavailable iron, it is fundamental to know the sources and fate of iron along the continental margins. We show that glaciers supply large quantities of Fe to the fjord environment, but the proportion of potentially bioavailable Fe in glacially derived material is low. While fjords were previously expected to reduce glacial iron delivery to the ocean^15^, we show that fjord sediments are a biogeochemically active interface between the land and ocean in which glacially sourced iron is transformed into potentially bioavailable Fe through benthic cycling. Our results show that surficial sediments near the fjord mouth are enriched in potentially bioavailable Fe that could be a source of iron to the marine shelf and open ocean environments, thereby promoting primary productivity. We furthermore highlight the sensitivity of benthic biogeochemical processes in fjord sediments, especially Fe-cycling, to glacial retreat and show that glacial retreat may reduce the sediments ability to serve as a source of iron to the overlying water column.

## Methods

### Field sites, sampling, and processing of samples

We sampled fjord sediment and particulate material from glacial sources in three fjords (Kongsfjorden, Lilliehöökfjorden, and Dicksonfjorden) located on the west coast of Spitzbergen, the largest island of the Svalbard archipelago (Fig. 1, Supplementary Table 5 and S6). For a more detailed description of the field, sites see supplemental information.

Fjord Sediment was sampled at 11 sites in Kongsfjorden and nine sites in Lilliehöökfjorden in June and July 2017, two sites in Lilliehöökfjorden in July 2018, and 5 sites in Dicksonfjorden in August 2018 aboard MS Teisten or MS Farm (Supplementary Table 5, Fig. 1). Sediment was retrieved with a Haps corer^62^ and sub-sampled aboard the ship using 2.8 cm (for SRR measurements) or 6 cm (for pore water and solid-phase geochemistry) diameter acrylic coring tubes. Sediment was stored at 4 °C until further processing within 2 days after sampling.

The glacial source material was sampled in Kongsfjorden in June and July 2017, and July 2018. In total, we collected seven pieces from individual icebergs with embedded sediment (Supplementary Fig. 2), four samples of glacial plume water in front of the KB/KV calving front, and six samples of meltwater from rivers along the southern and northern shore of Kongsfjorden (Supplementary Table 6, Fig. 1). The material from the meltwater rivers was collected directly at their mouth before entering the fjord. Material from the Dicksonelva plume at the head of Dicksonfjorden was sampled in August 2018 (Supplementary Table 6).

The distances of the stations relative to the main glacial source was determined by geospatial analysis using qGIS (v. 3.10). We used the imagery seen in Fig. 1 to measure the distance from the glacier terminus to the GPS determined sample point. The imagery was collected ~1 month after our samples were collected and represented the glacial terminus at the time of sample collection.

### Processing and subsampling of sediment cores

The 6-cm wide subcores were sliced in an anoxic glove bag under N_2_ atmosphere (<0.5% atmospheric O_2_ concentration, monitored continuously with a trace-range optical oxygen sensor TROXROB10 connected to a Firesting O_2_ -meter, Pyroscience). The cores were processed outside the laboratory at ambient temperature (4–8 °C) inside an anoxic glove bag as described in detail by Michaud et al.^31^. All plasticware used for subsampling was made anoxic by placing the plasticware and an oxygen scrubber (AnaeroGen, ThermoFischer) in a heat-sealed gas-tight plastic bag (Escal Neo, high gas barrier bag, Mitsubishi Gas Chemical Co., Inc.) for at least 24 h. The sediment cores were sliced into 1–3 cm sections down to a depth of 13 cm. After each section was homogenized, subsamples of sediment were taken for (i) Fe extractions, (ii) determination of porosity, water amount, TOC and TN, and (iii) pore water geochemistry. The subsamples for Fe extractions, porosity, water content, TOC, and TN were immediately frozen at −20 °C. After closing the centrifuge tubes inside the glove bag under N_2_ atmosphere, the pore water samples were centrifuged for 15 min at 3000 × *g* outside the glove bag. The tubes were immediately returned to the glove bag after centrifugation, and the supernatant was filtered by centrifugation (5 min, 14,100 × *g*) in spin filters (0.45 µm nylon membrane, Norgen Biotek). For dissolved Fe(II) and Mn analysis, an aliquot of the filtrate was acidified (HCl, 1 M final concentration) and the remaining was used for sulfate quantification. All pore water samples were stored at 4 °C in the dark until analysis.

### Processing and subsampling of glacial source material

Particulate material was extracted from plume and river-water by centrifugation (15 min., 3000 × *g*). Samples of sediment-loaded icebergs were first rinsed with milliQ water on the exposed surfaces, then we let the ice melt inside a clean plastic bag before centrifugation. In all cases, the pellets were collected and frozen at −20 °C until analysis.

### Pore water chemistry

Dissolved Fe(II) and Mn in the pore water were measured spectrophotometrically by the ferrozine assay^63^ and the formaldoxime assay, respectively. The formalodxime assay was adapted according to Otte^64^ to exclude interference from high Fe^2+^ concentration in the sample. Both assays were performed in 96-well plates and the absorbance was measured at 562 nm for Fe(II) and 450 nm for Mn with a plate reader (FLUOstarOmega, BMG Labtech). Sulfate concentration in pore water was quantified on 1:100 diluted samples using suppressed ion chromatography (Dionex).

### Sulfate reduction rate measurements

Sulfate reduction rates (SRR, nmol cm^−3^ d^−1^) were determined by injecting ^35^SO_4_^2−^ into intact, 20–25 cm long, 2.8 cm diameter sediment cores^65^. Fifty kBq of carrier-free ^35^S-SO_4_^2−^ was injected at 1-cm depth intervals through ports sealed with polyurethane-based elastic sealant (Sikaflex^®^−11FC^+^, Sika)^66^. After 10–14 h of incubation at near in situ temperature (2 °C), the cores were sliced in 1-cm sections, which were added immediately to 10 ml of 10% zinc acetate and homogenized by vortexing. The zinc acetate-fixed samples were stored at −20 °C until analysis. The cold chromium method^66^ was used to separate radiolabeled total reduced inorganic sulfur (TRIS) from the sample and the evolved H_2_S was trapped as Zn^35^S in 5 mL of 5% zinc acetate solution. Scintillation counting was used to analyze the radioactivity in the sulfate and TRIS pools and sulfate reduction rates were calculated according to Jørgensen^65^. To determine the water content and porosity of the sediment, required for calculation of SRR, the weight loss of a known volume of sediment after drying to constant weight at 105 °C was determined. Some of the SRR data is already published in a recently accepted manuscript^67^. For which stations this is the case is stated in Supplementary Table 5.

### TOC and TN analysis

For TOC and TN measurements, sediment was dried at 105 °C and powdered using a planetary micro mill (Pulverisette 23, Fritsch). After acidification with HCl to remove inorganic carbon and washing steps with MQ water to remove additional salt from the HCl, the powdered sediment was dried again and the carbon and nitrogen content and isotopic composition were measured with an elemental analyzer (Thermo Fisher Scientific Flash EA 1112) coupled to an IRMS.

### Sequential endpoint Fe extractions

Sequential endpoint extractions with HCl, to separate the poorly crystalline (0.5 M HCl, 1 h, 20 °C) from the crystalline (6 M HCl, 24 h, 70 °C) Fe(II) and Fe(III) in the sediments and the glacial source samples, were done as described by Laufer et al.^26^. Fe(II) and total Fe concentrations in the extracts were determined spectrophotometrically by the ferrozine assay^63^. For total Fe concentrations, all Fe(III) was reduced to Fe(II) with the reductant hydroxylamine hydrochloride (HAHCl, 10% w/v in 1 M HCl) before the assay. Fe(III) was calculated from the difference between Fe(II) and total Fe concentrations.

### Ascorbate time-course extractions

Ascorbate extractable Fe (FeA) was determined in ascorbate Fe reduction time-course extractions^42,43^. These experiments were conducted in 100 ml Schott bottles, equipped with four-port Schott Duran® Pressure Plus screw caps. Of the screw caps, two ports were used for flushing the bottles with N_2_. One port was used for the injection of the extraction solution. These three ports were equipped with tygon tubings and three-way valves. The last port was used for fluid sampling during the extraction and was equipped with a Rhizon soil moisture sampler (0.15 µm pore size, Rhizosphere Research Products). This setup allowed us to follow the time-dependent dissolution of Fe during the extractions

Inside an anoxic glove bag, the frozen sediment was thawed for the extractions (O_2_ < 0.5% atmospheric concentration). Between 0.5 and 1 g of sediment was weighed into the Schott bottles, the bottles were closed inside the anoxic glove bags and immediately flushed with N_2_ after they were taken out of the glove bag. After 3 min of flushing with N_2_, 100 ml of extraction solution, containing 0.6 M sodium bicarbonate, 0.17 M sodium citrate, and 0.1 M sodium citrate, pH 7.5 was added through one port. Stirring with a Teflon-coated magnetic stir bar was started and continued throughout the extraction. Immediately after adding the extraction solution, samples for determining the dissolved Fe(II) concentration were taken through the Rhizon and fixed in 1 M HCl (final concentration). Sampling continued in intervals with increasing length, starting with 5 min at the beginning, increasing to 10, 20, and 30 min, and finally every 1–2 h within the first 400–500 min. Sampling was continued the next day until a stable Fe(II) concentration was reached (which was the case after a maximum 32 h). Fe(II) concentrations were quantified using the ferrozine assay as described above for the pore water samples.

A reactive continuum model (ref. ^42^, Eq. 1) was used to calculate the amount of extractable iron (M_(0)_ µmol g dw^−1^), the reducibility of FeA (v/a, s^−1^), the composition of FeA (1 + 1/v), and lability of FeA (initial rate of reduction, µmol g dw^−1^ s^−1^) as described in detail by Laufer et al.^26^ (Eq.1, Table 1).

1 $$\frac{{\mathrm{J}}}{{{\mathrm{M}}_{\left( 0 \right)}}} = \frac{{\mathrm{v}}}{{\mathrm{a}}}\left( {\frac{{{\mathrm{M}}_{\left( {\mathrm{t}} \right)}}}{{{\mathrm{M}}_{\left( 0 \right)}}}} \right)^{1 + \frac{1}{{\mathrm{v}}}}$$

The other parameters in Eq. 1, are: J, the dissolution rate (µmol g dw^−1^ s^−1^) and M_(t)_, which is the ascorbate extractable Fe(III) left in the sediment at time t (µmol g dw^−1^). The time-dependent development of M_(t)_ is described by Eq. 2:

2 $${\mathrm{M}}_{({\mathrm{t}})} = {\mathrm{M}}_{(0)}\left( {\frac{{\mathrm{a}}}{{{\mathrm{a}} + {\mathrm{t}}}}} \right)^{\mathrm{v}}$$

We defined M_(0)_ as the maximum amount of Fe released into the solution at the end of the extraction. Using Eq. 2, the parameters a and v were fitted to Eq. 2 by applying the nonlinear least squares (nls) function in R. The parameters v/a and 1 + 1/v were calculated based on the fitted v and a. The amount of Fe dissolved at any time point (Fe_d_ (t) in µmol g dw^−1^) was calculated according to Eq. 3, in order to visualize the model results of the release of Fe(II) into the solution over time.

3 $${\mathrm{Fe}}_{\mathrm{d}}\left( {\mathrm{t}} \right) = {\mathrm{M}}_{(0)} - {\mathrm{M}}_{({\mathrm{t}})}$$

The initial rate of Fe(II) dissolution per g dw^−1^ (reducibility of FeA) was calculated by multiplying the apparent rate constant v/a with M_(0)._

Statistical tests on the significance of the increase in M_(0)_ and initial rates in the surface sediment with increasing distance from the glacial source were performed by linear regression analysis in R.

The R code for these calculations can be found in a github repository (https://github.com/klaufer-meiser/Time-course_extractions_code).

### Microbial time-course extractions

A pure culture of *Shewanella frigidimarina* DSM-12253^68^ was used to determine microbially reducible Fe (FeM) in time-course extractions. Pre- cultures of *S. frigidimarina* were grown aerobically on full-strength Luria-Bertani (LB) medium, where NaCl was exchanged with artificial seawater (ASW) salts (NaCl, 27.5 g l^−1^; MgCl_2_*6 H_2_O, 5.38 g l^−1^; MgSO_4_*7H_2_O, 6.78 g l^−1^; KCl, 0.72 g l^−1^; CaCl_2_*2H_2_O, 1.4 g l^−1^; NH_4_Cl, 1 g l^−1^; K_2_HPO_4_, 0.05 g l^−1^). From one colony of the LB plate, an overnight culture was grown in 10 ml liquid LB ASW at 20 °C. From the actively growing overnight culture, larger volumes (200-300 ml) of liquid LB-ASW were inoculated and grown overnight to an OD of 2–2.5, which meant the cultures were in late exponential phase (which was the case after ca. 16 h). For the experiments, concentrated *S. frigidimarina* cultures were prepared by washing the cultures three times with ASW by centrifuging (20 min, 3000 × *g*), after each centrifugation the supernatant was discarded and the pellet was resuspended in fresh ASW. Next, the culture was re-suspended in ASW in a serum vial to a cell density of ca. 3 × 10^10^ cells ml^−1^ and the concentrated culture was made anoxic by bubbling with sterile-filtered N_2_ (0.22 µm pore size).

Inside an anoxic glove bag (N_2_ atmosphere, O_2_ < 0.5% atmospheric concentration) between 0.1 and 0.4 g of the sediment was weighed into 15 ml Hungate tubes and the tubes were closed with a butyl stopper and screw cap. Afterwards, 9 ml of anoxic ASW, amended with Na-bicarbonate buffer (22 mM), Na-citrate (170 mM), Na-lactate (10 mM) and Na-molybdate (20 mM), adjusted to pH 7.5, was added. The Headspace was flushed with N_2_/CO_2_ (80:20). Then, 1 ml concentrated *S. frigidimarina* culture was added to reach a final cell concentration of ca. 3 × 10^9^ cells ml^−1^. All extractions were performed in triplicates while a fourth replicate remained as an uninoculated control and served as a measure of native Fe(III)-reducer population activity in the sediment.

The microbial extractions were performed on a horizontal shaker at 20 °C in the dark, to prevent photoreduction. Sampling was performed with a N_2_-flushed syringe with a thick needle (0.5 × 25 mm). One-hundred µl of the sample were added to 900 µl of 1 M HCl and acid-extraction was performed for 1 h on a horizontal shaker. The acid-extraction was stopped by centrifugation (5 min, 12,100 × *g*) and the supernatant was used for Fe(II) analysis with the ferrozine assay^63^. Samples were taken directly before and after the addition of the *S. frigidimarina* culture and the Fe(II) concentration was followed over time until it reached a constant value. Sampling intervals were between 10 min and 300 h, and were chosen depending on the observed Fe(II) reduction rates. Uninoculated controls were sampled in parallel throughout the duration of the experiment.

The parameters M_(0)_ (amount of FeM), v/a (lability of FeM), 1 + 1/v (the composition of FeM) and initial rates (reducibility of FeM) were determined with the same equations as described above for the ascorbate time-course extractions.

### Particle size analysis

Particle size analysis was performed at Binghamton University’s Analytical and Diagnostics Laboratory. Approximately 1 g of bulk sample was gently disaggregated and treated for the removal of organic matter with ~20 ml of 27% hydrogen peroxide (H_2_O_2_) in hot water bath. Smear slides of treated samples were analyzed under a binocular microscope in order to assess for the presence of biogenic components. As no biogenic material could be detected on the smear slides, samples were then treated with 10% sodium hexametaphosphate and shaken for 12 h prior to analysis on the Beckman Coulter LS 13320 Laser Diffraction Analyzer.

### ^57^Fe Mössbauer spectroscopy

Mössbauer spectroscopy analysis was performed at the Center for Applied Geosciences at the University of Tübingen. Freeze-dried samples were loaded into Plexiglas holders (area 1 cm^2^), forming a thin disc, within an anoxic glovebox (100% N_2_). Sample holders were transported to the instrument within airtight bottles which were only opened immediately prior to loading into a closed-cycle exchange gas cryostat (Janis cryogenics) under a backflow of He to minimize exposure to ambient air. Spectra were collected at 77 and 5 K using a constant acceleration drive system (WissEL) in transmission mode with a ^57^Co/Rh source. All spectra were calibrated against a 7-µm thick α-^57^Fe foil that was measured at room temperature. The analysis was carried out using Recoil (University of Ottawa) and the Voigt Based Fitting (VBF) routine^69^. The half width at half maximum (HWHM) was constrained to 0.138 mm s^−1^ during fitting.

## Supplementary information

Supplementary information

## Data Availability

The authors declare that all the data supporting the findings of this study and all source data are available in the article and its Supplementary Information file. Any further information is available from the corresponding author upon request.

## Code availability

The R code used for calculating parameters of the kinetic extractions can be found in a github repository (https://github.com/klaufer-meiser/Time-course_extractions_code)^70^.
