# Supplementary material for: Potentially bioavailable iron produced through benthic cycling in glaciated Arctic fjords of Svalbard
Source: Nat Commun. 2021 Mar 1;12:1349. doi: 10.1038/s41467-021-21558-w (PMC7921405; doi:10.1038/s41467-021-21558-w)
Supplement: Supplementary file 1 — Supplementary information [file 41467_2021_21558_MOESM1_ESM.pdf]

Potentially bioavailable iron produced through benthic cycling in glaciated Arctic fjords of Svalbard

Authors:

Katja Laufer-Meiser[*1,2], Alexander B. Michaud[1,3], Markus Maisch[4], James M. Byrne[4,5], Andreas Kappler[2,4], Molly O. Patterson[6], Hans Røy[1], Bo Barker Jørgensen[1]

[1] Center for Geomicrobiology, Department of Biology, Aarhus University, Denmark

[2]Now: GEOMAR, Helmholtz Centre for Ocean Research Kiel, Germany

[3]Now: Bigelow Laboratory for Ocean Sciences, Maine, USA

[4]Center for Applied Geosciences, University of Tübingen, Germany

[5]Now: School of Earth Sciences, University of Bristol, Wills Memorial Building, Queens Road, Bristol BS8 1RJ, United Kingdom

[6]Department of Geological Sciences and Environmental Studies, Binghamton University, New York, USA

[*]**Corresponding Author**

Katja Laufer-Meiser. Wischhofstraße 1-3, 24148 Kiel, Germany. Tel.: 0049 431 600 1267. E-mail: klaufer@geomar.de

Supplementary Information

Field site description

Kongsfjorden (KF) is oriented south-east to north-west, 20 km long and 4-10 km wide. The inner basin, with water depths < 100 m, extends over 10 km from the front of several marine-terminating glaciers [1,2]. The outer basin has a maximum water depth of 394 m. Along its east and north coast, Kongsfjorden has five tidewater glaciers, Kronebreen (KB) and Kongsvegen (KV) (converging approx. 5 km up-glacier from their terminus), Kongsbreen, Conwaybreen, and Blomstrandbreen, as well as several smaller land-terminating glaciers on its south coast. The KB/KV glaciers override Fe-rich Devonian red sandstone beds [3–6] and deliver high amounts of red Fe-rich suspended material to Kongsfjorden, which produces red, Fe-rich fjord sediments (Figure 1)[7–9]. As the KB/KV glaciers deliver by far the highest amount of detrital material to Kongsfjorden[7], they can be considered the main glacial source in Kongsfjorden. ***Lilliehöökfjorden (LF)*** is the western arm of Krossfjorden and is oriented north to south. Krossfjorden in total is 30 km long and 4-6 km wide, the maximum water depth is 375 m and it merges with Kongsfjorden in the south [2]. Lilliehöökfjorden has one main glacier at its head, Lilliehöökbreen (LB). Besides LB, Lilliehöökfjorden has no major glacial sources. About 15 km away from the calving front of LB, Llilliehöökfjroden and Möllerfjorden merge. Further south from the Llilliehöökfjorden/Möllerfjorden merging point, there are several smaller land and marine-terminating glaciers in Krossfjorden. The bedrock of Lilliehöökbreen mainly consists of medium-grade metamorphic rocks comprising of marbles, mica-schists, and minor amounts of quartzites of Middle Proterozoic age[5], which produce greyish sediments in the fjord, visually very different from Kongsfjorden sediment (Figure 1 and S5). ***Dicksonfjorden (DF)***, a side arm of Isfjorden, is located on the west coast of Svalbard further south than Kongsfjorden and Lilliehöökfjorden. It is 28 km long and 4- 7 km wide. The maximum water depth is 123 m [11] (http://toposvalbard.npolar.no). The main glacial source of Dicksonfjorden is the pro-glacial river Dicksonelva, running through Dicksondalen before entering Dicksonfjorden in a large river delta with a tidal mudflat at is head. Dicksonelva is fed by several smaller glaciers, of which Battyebreen is the largest. The Dicksonfjorden glaciers mainly override Fe-rich Devonian red sandstone [3–6], similar to the KB/KV glaciers in Kongsfjorden. The sediment in Dicksonfjorden is red and apparently iron rich, similar to Kongsfjorden. The ***bottom waters*** in all three studied fjords are oxic.

^{57}Fe Mössbauer spectroscopy results and interpretation

All Mössbauer spectra, collected at 77 K, generally revealed two very clearly distinct two-line sites (doublets, Db; Supplementary Figure 1). The hyperfine field parameters of Db1 showed a relatively high quadrupole splitting (ΔE_Q) ranging from ΔE_Q = 2.78 to 2.83 mm/s and a center shift (CS) of CS = 1.22 to 1.24 mm/s in all spectra. These parameters are indicative for high spin Fe(II) mineral compounds. The narrow doublet (Db2) were fitted with much smaller values for CS ranging from 0.47 to 0.52 mm/s and a relatively low ΔE_Q from 0.52 to 0.80 mm/s. These hyperfine parameters are indicative for paramagnetic, poorly crystalline Fe(III) mineral phases such as ferrihydrite or lepidocrocite although the presence of Fe(III) within e.g. phyllosilicates cannot be excluded [12]. Except for samples LF1 0-1 cm and LF5 0-1 cm, the collected spectra at 77 K showed the presence of a magnetically-ordered iron mineral compound indicated by the wide sextet (Sxt) forming in the spectra (Supplementary Figure 1). The hyperfine parameters of this Sxt showed relatively consistent values with a CS = 0.45 to 0.48 mm/s and a wide hyperfine field (B_{hf}) of more than 50 T in samples from KF. This wide hyperfine field is indicative for the presence of a more crystalline Fe(III) mineral phase such as hematite.

Spectra from samples Plume KB a and IB KB b reveal that the composition of iron mineral phases differ considerably between these two samples. While the sample from Plume KB a showed a relative abundance for the higher crystalline Fe(III) mineral phase of only 17.8±1.6 %, the sample IB KB b showed a two-fold higher abundance for this more crystalline mineral phase of 41.3±1.9% (Supplementary Table 2). Also, the relative abundance for Fe(II) and Fe(III) of the non-magnetically ordering poorly crystalline iron mineral phases differ slightly between the samples with 58.9±0.3 % Fe(II) in Plume KB a and only 31.6±2.9 % in IB KB b.

Samples from KF sediment not only differ in their relative Fe(II)/Fe(III) ratios but also in the relative abundance for different iron mineral phases. Sample KF1 0-1 cm showed 63.2±0.7% of a non-magnetically ordered Fe(II) mineral phase and only 15.7±0.8% of a poorly crystalline Fe(III) mineral compound. The more crystalline mineral phase (likely to be hematite) accounts for 21.1±1.3% of the relative spectral area in the sample collected at KF1 0-1 cm. In contrast, the collected spectrum of sample KFa7 0-1 cm demonstrates that only 10.3±2.1% of the relative abundance can the attributed to this higher crystalline phase. The relative amount of Fe(III) was 41.0±1.1% while the poorly crystalline phases had relative abundance of 48.7±0.3% in sample KFa7 0-1 cm.

Samples from LF sediment showed a relatively similar iron mineral phase composition with a considerably higher abundance of Fe(II) mineral phases which accounted for 80.4±0.7% and 72.5±0.4% of the spectral area for samples LF1 and LF5, respectively. The spectra of these samples did not indicate the presence of a magnetically-

ordered mineral phase at 77 K as was observed in all other samples. The main components in samples from LF are considered to be paramagnetic Fe(II) and Fe(III) mineral phases.

The difference in the iron mineral composition between the individual samples was also recognizable in the streak color of the individual sample material (Supplementary Figure 5). While the two source (plume & iceberg) samples and samples from KF showed a very dominant red/brownish color, samples from LF do not show an intensive red streak color. This is supports the aforementioned interpretation that the higher crystalline and magnetically-ordered phase (which is likely to be hematite) is only present in samples KF1 0-1 cm, KFa7 0-1 cm, Plume KB a and IB KB b, while samples LF1 0-1 cm and LF5 0-1 cm only contain a composition of paramagnetic (and potentially poorly crystalline) Fe(II) and Fe(III) phases.

Uncertainty in mineral identities identified by Mössbauer spectroscopy. The 77 K spectra suggest that samples from Plume KB a differ mainly in their relative abundance of hematite. With significantly more hematite in the IB KB b sample compared to Plume KB a. The 5 K spectra of these samples support the presence of twice as much hematite in the IB KB b sample (Supplementary Figure 1).

The 5 K spectra of KF sediment samples support the hypothesis that the KF1 0-1 cm sample contains twice as much hematite than sample KFa7 0-1 cm (Supplementary Figure 9, Supplementary Table 2). The hyperfine parameters of the doublets do not closely match literature values, although they potentially correspond to the presence of mixed valent Fe(II)/(III) bearing phyllosilicates. A poorly ordered sextet was required to accurately fit the spectrum. The identity of this phase is unknown.

Paramagnetic relaxation was also detected in the 5 K spectra of samples LF1 0-1 cm and LF5 0-1 cm. Furthermore, the absence of a wide sextet with a hyperfine field >50 confirms that hematite was not present in samples from LF sediment. However, the similarity of the 5 K spectra and the same fitting parameters suggest that samples LF1 0-1 cm and LF5 0-1 cm are very similar in terms of their iron mineral composition, with the exception of hematite

Grain size analysis and interpretation

Over 95% of the grain size distributions from surface sediment samples recovered along three transects are characterized by silt and clay (< 63 μm or 4 ϕ) (Supplementary Figure 6). Through dynamic processes relating to sediment erosion, deposition and aggregate breakup, silt material 63-10 μm (4 – 6.6 ϕ) will undergo sorting in

response to hydrodynamic processes, whereas, fine silt size particles < 10 μm (6.6 ϕ) behave in a cohesive manor in the same way as clay particles (< 2 μm or > 9.0 ϕ). Thus, sorting of silt maybe observed through current winnowing or as the distance from areas of higher energy (i.e., rivers and glacial meltwater plumes) increases with the finer grain component accumulating farther away. Detailed grain size analysis of the lithogenic component along three transects demonstrate no systematic relationship between the percent fine grain material (< 63 μm or > 4 ϕ) and the distance from glacier source (Supplementary Figure 6). While there are clear differences between grain size distributions from source material, surface sediment from stations do not systematically get finer grained in texture as distance increases. For example, across three transects, sites farthest from the glacier sources nearly overlap and appear indistinguishable from or contain a slightly higher percent (at most ~2-1 %) of coarser silt material, than adjacent sites located closer to the glacier. Thus, the increase of FeR over distance from the glacier cannot be explained as a function of the transport of the fine grain particles.

Changes of SRR over distance in relation to changes TOC and C:N over distance from the glacial source

The increasing amount of TOC and decreasing C:N ratios with greater distance from the fjord heads in all three investigated fjords (Figure 6, Supplementary Table 7), indicate the presence of more fresh and labile organic carbon further out in the fjord. We therefore expected that organic carbon mineralization in the fjord sediment, and thereby also SRR, would follow this pattern and increase with increasing distance from the fjord head. However, in all four investigated fjord transects, SRR at the station furthest away from the head of the fjord were not much higher compared to the station closest to the fjord head (Figure 6 and S8). In KFa and KFb, SRR peaked mid-fjord but then also deceased to a value close to the starting value again. This pattern indicates a change from a predominance of SR close to the fjord head, where there is only little FeA and FeM, towards a higher proportion of Fe reduction further away from the fjord head. This is likely driven by the higher content and reactivity of FeA and FeM further away from the glacier (Supplementary Figure 4, Supplementary Table 8 and S9).

In Kongsfjorden, highest SRR were measured at station KFb3 (up to 330 nmol cm^{-3} d^{-1}; Supplementary Figure 8), also integrated rates of SRR were highest (Figure 6) and high TOC values were measured. The high SRR are probably connected to the close by bird cliff, where a colony of kittiwakes are nesting [13,14]. The large bird

colony contributes to a higher primary productivity on land around the colony, likely introducing elevated amounts of nutrients and organic carbon to the close by marine environment.

At KF1, KFa3, KFa5, KFb3 SRR peaked directly at the sediment surface (Supplementary Figure 8). At stations further away from the Kongsfjorden head, the peak in SRR was found deeper down in the sediment. Depth-integrated rates of SR at first increased with increasing distance from the fjord head but then dropped again (Figure 6). In Lilliehöökfjorden maximum SRR were 80 nmol cm^{-3} d^{-1} and no surface peaks were found, but SRR reached a maximum between 3 and 8 cm sediment depth (Supplementary Figure 8). In Dicksonfjorden, the maximum SRR was 6 nmol cm^{-3} d^{-1} and no substantial change of depth-integrated SRR was found over distance from the fjord head (Figure 6).

Supplementary Figures

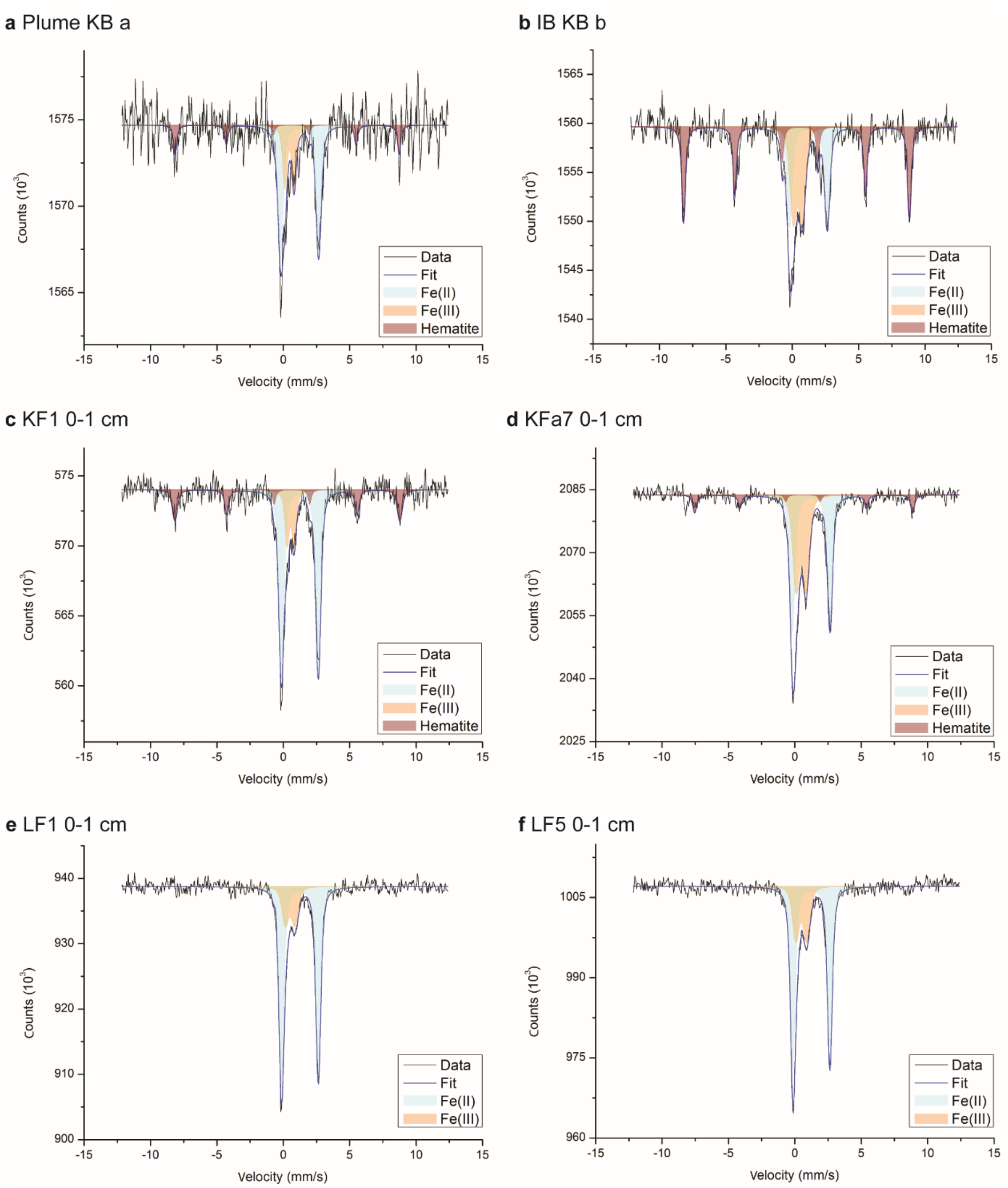

Supplementary Figure 1 – Mössbauer spectra collected at 77 K. All samples showed a wide doublet (Db1; light blue) that can be attributed to the presence of a high-spin Fe(II) mineral phase in all samples. The narrow doublet (Db2; orange) in all samples can be attributed to the presence of a non-magnetically ordered poorly crystalline Fe(III) mineral phase similar to ferrihydrite. Samples a)-d) showed an additional sextet feature (red) that shows the presence of a magnetically-ordered mineral phase at 77K. The wide hyperfine field (>50 T) suggests hematite as iron(III) oxide likely to be present in these samples.

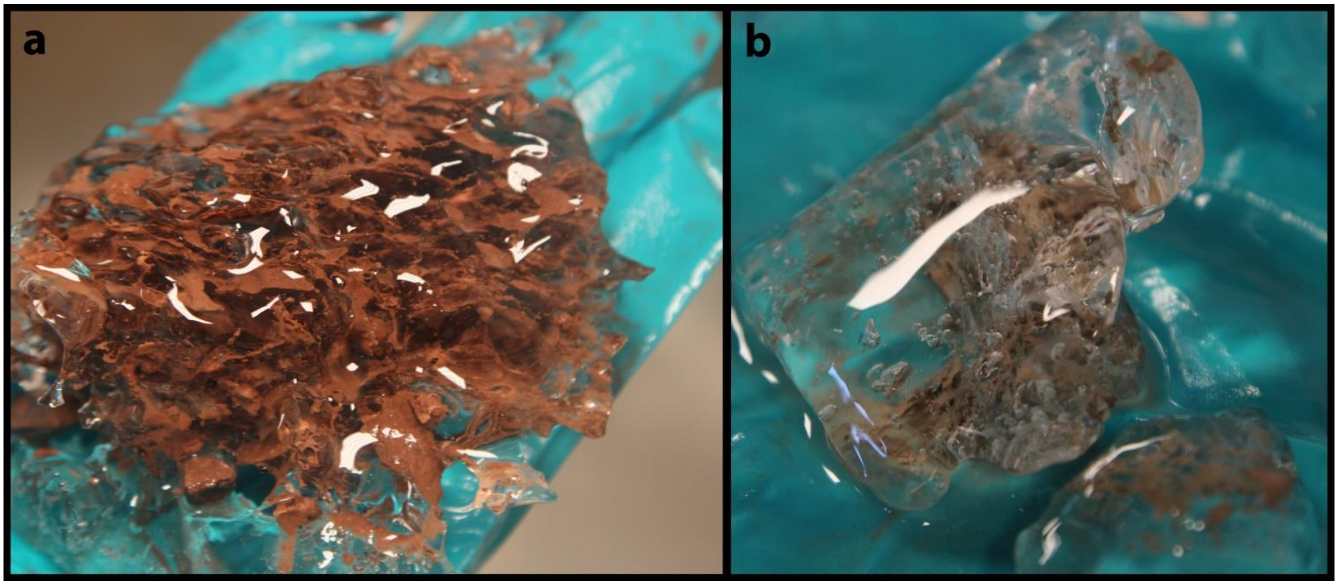

Supplementary Figure 2: Small iceberg samples: a) IB KB from in front of Kronebreen, b) IB KV from the front of Kongsvegen.

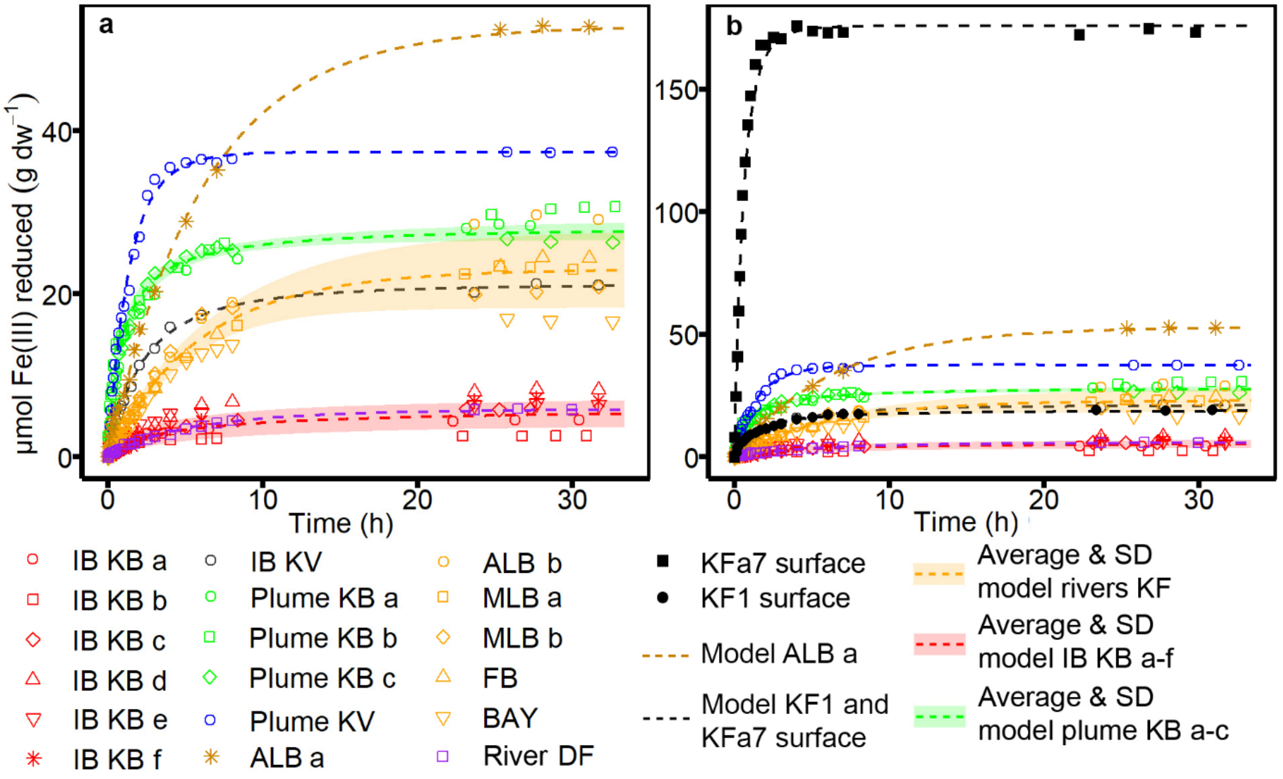

Supplementary Figure 3: Labile iron characteristics from ascorbate extractions of glacial source material and sediment. **a** Dissolution curves of different glacial sources from Kongsfjorden and the plume sample from Dicksonfjorden. **b** ascorbate extractions of the surface sediment of the station closest to the main glacial source (KF1) and furthest away from the main glacial source (KFa7) compared to the same sources as shown in **a**. Note the difference in y-axis scale between panel **a** and **b**. Symbols show the measured dissolution curves while the models are indicated by dashed lines. If several replicates of one type of source were measured, which were very similar, the average and SD (shaded area) of the models for these samples is shown. IB KB = iceberg samples from in front of the Kronebreen glacier in Kongsfjorden (red). IB KV = iceberg sample from in front of the Kongsvegen glacier in Kongsfjorden (dark grey). Plume KB = samples from the proglacial plume in front of the Kronebreen glacier (light green). Plume KV= sample from the proglacial plume in front of the Kongsvegen glacier (blue). ALB= Austre Lovenbreen; MLB = Midre Lovenbreen; FB= Ferringbreen; BAY= Bayelva river; all in KF (ocher). The average of Kongsfjorden rivers includes ALB b, MLB a, MLB b, FB and BAY. River DF = sample of the plume of Dicksonelva at the head of Dicksonfjorden (purple). Details about sampling locations and dates can be found in Supplementary Table 9.

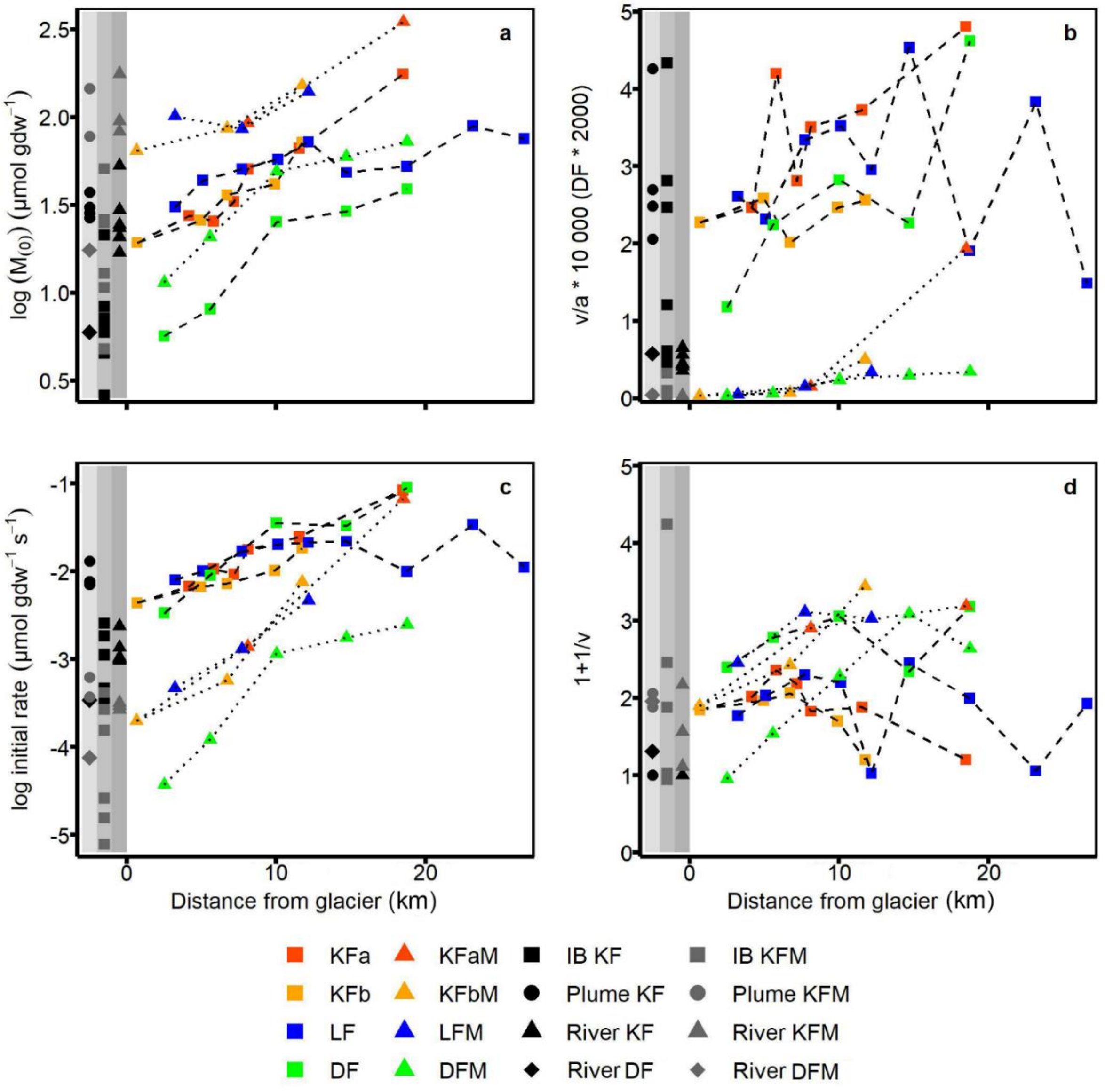

***Supplementary Figure 4: Amount ($M_{(0)}$), reducibility (apparent rate constant, v/a), lability (initial rate), and composition (1+1/v) of FeA and FeM over distance from the glacial source**.* Glacial sources are shown in the grey shaded areas: light grey=samples of proglacial plumes and meltwater rivers at the head of the fjord, medium grey= icebergs, dark grey = meltwater rivers at the side of the fjord.

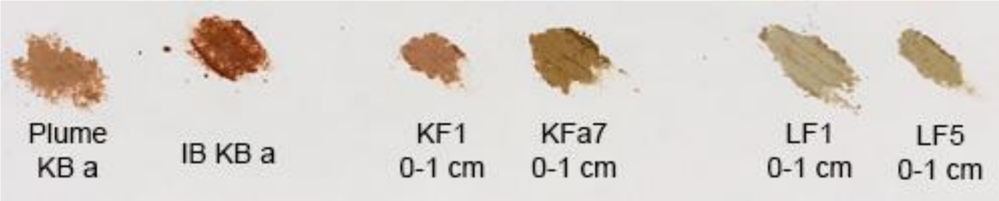

Supplementary Figure 5 – Streak color of individual samples.

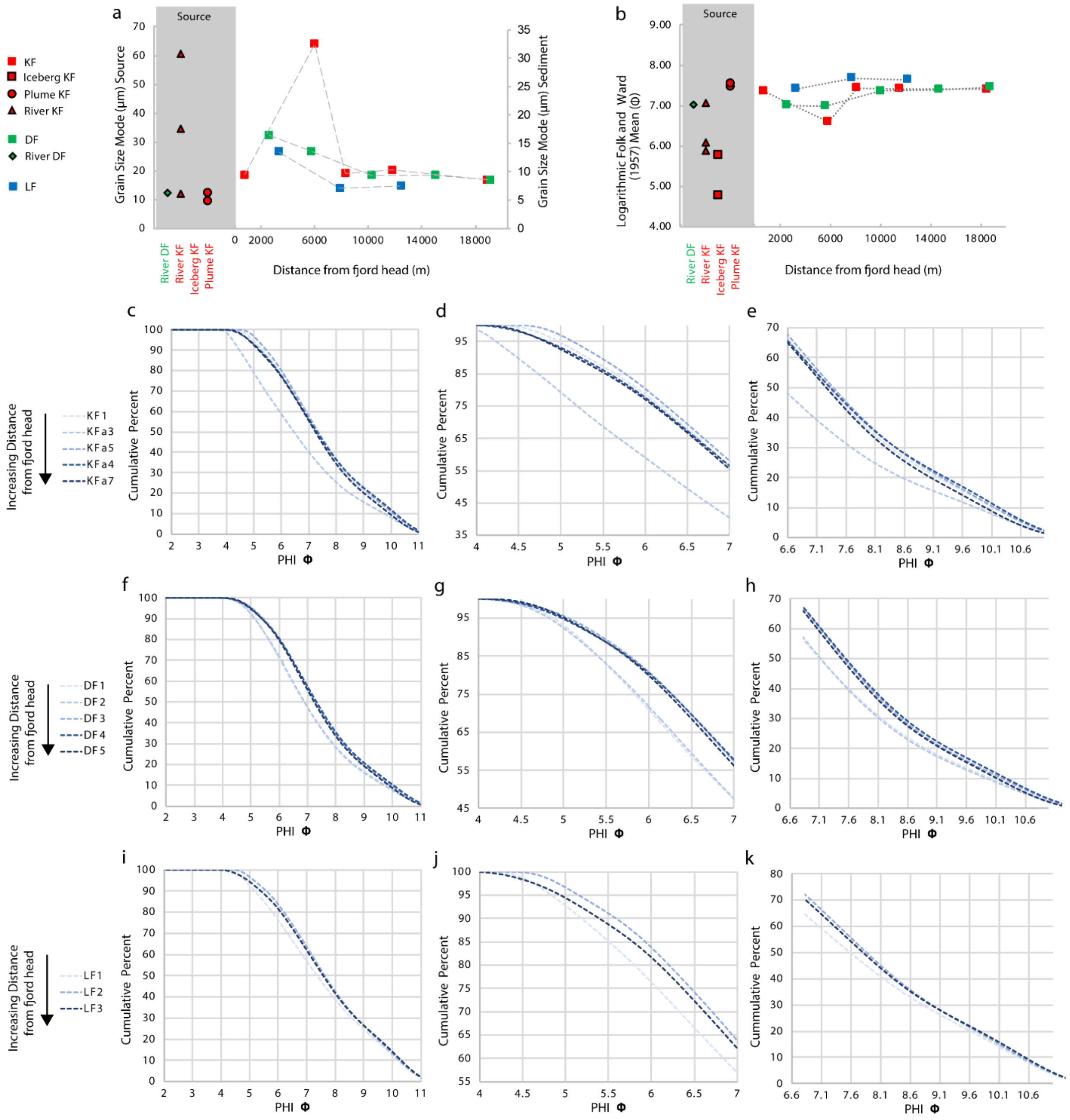

Supplementary Figure 6: Results from particle size analysis of surface sediment and glacial sources. **a** shows the mode of particle size distribution (PSD) over distance from fjord head for the sediment samples from all three fjords as well as the sources (grey area). **b** shows mean Φ value of the PSD over distance for the sediment samples from all three fjords as well as the sources (grey area). **c-k** show the distribution of Φ as cumulative percent in the surface sediments of all three fjords for the entire PSD (2-11 Φ), silt material that undergoes size sorting (4 – 6.6 Φ), and fine silt and clay that behaves in a cohesive manor (>6.6 Φ).

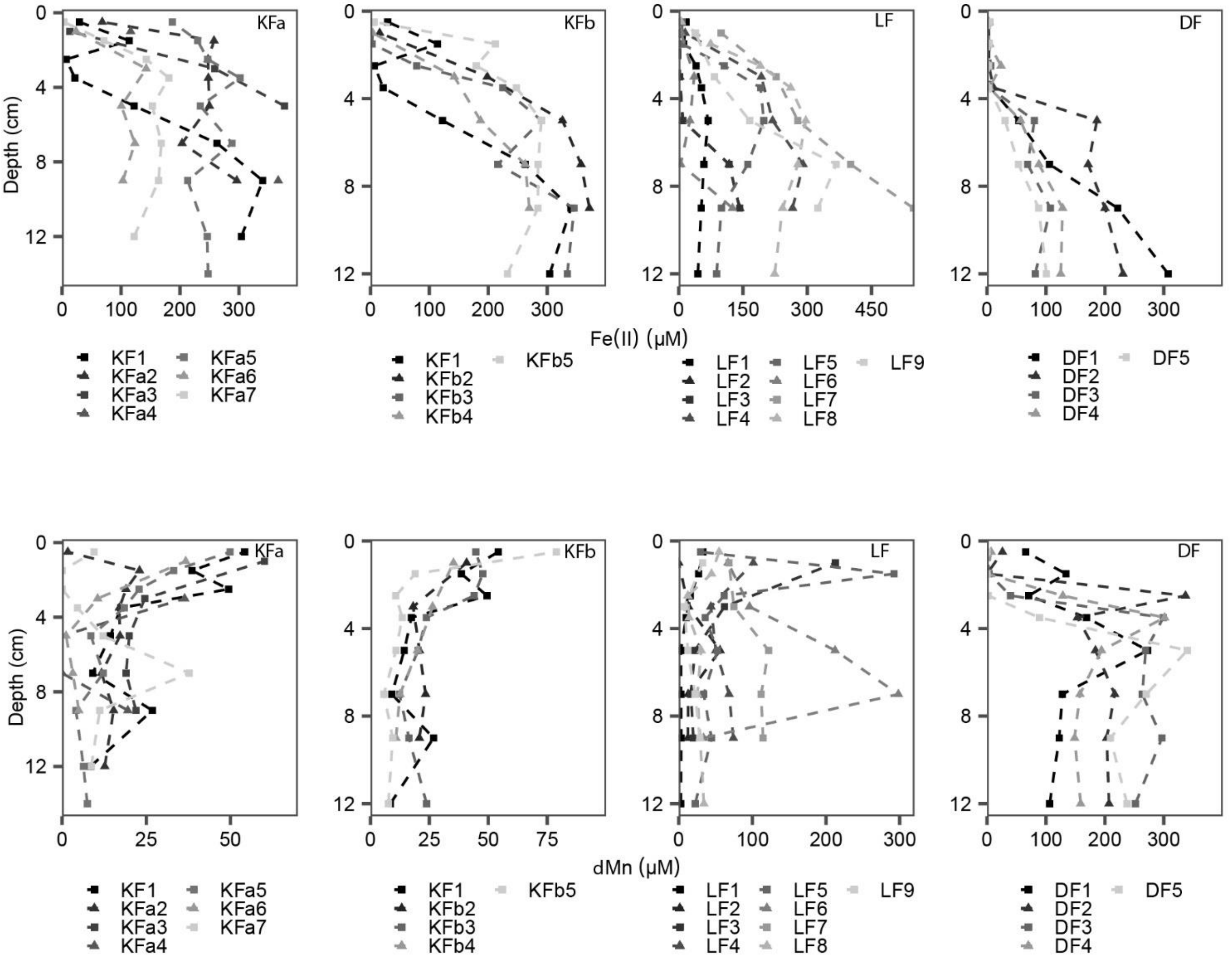

Supplementary Figure 7*: *Pore water dissolved Fe(II) and dissolved Mn (dMn) profiles per transect over depth.

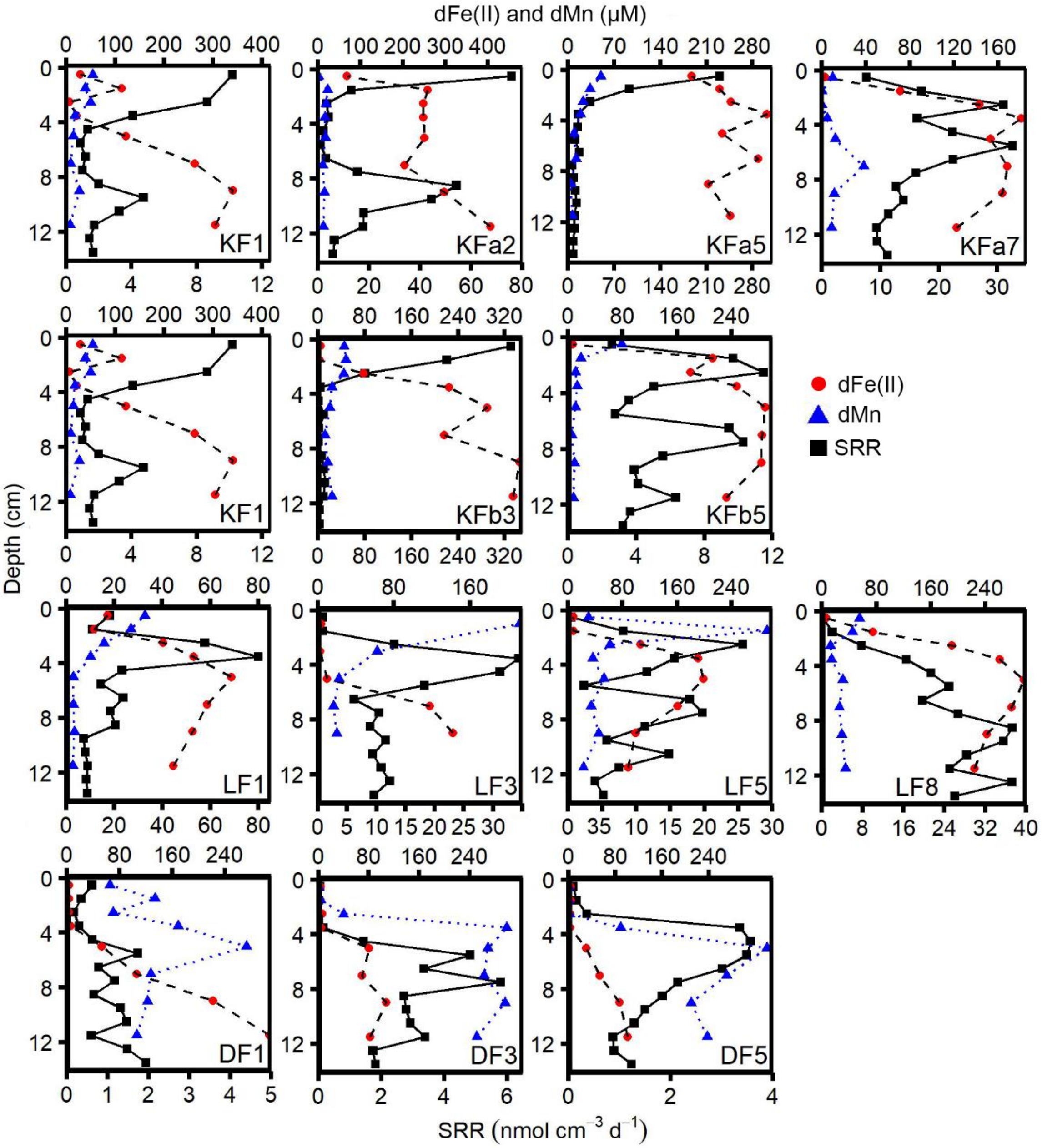

Supplementary Figure 8: Pore water profiles of dFe(II) and dMn and distribution of sulfate reduction rates (SRR) at the main stations in all transects.

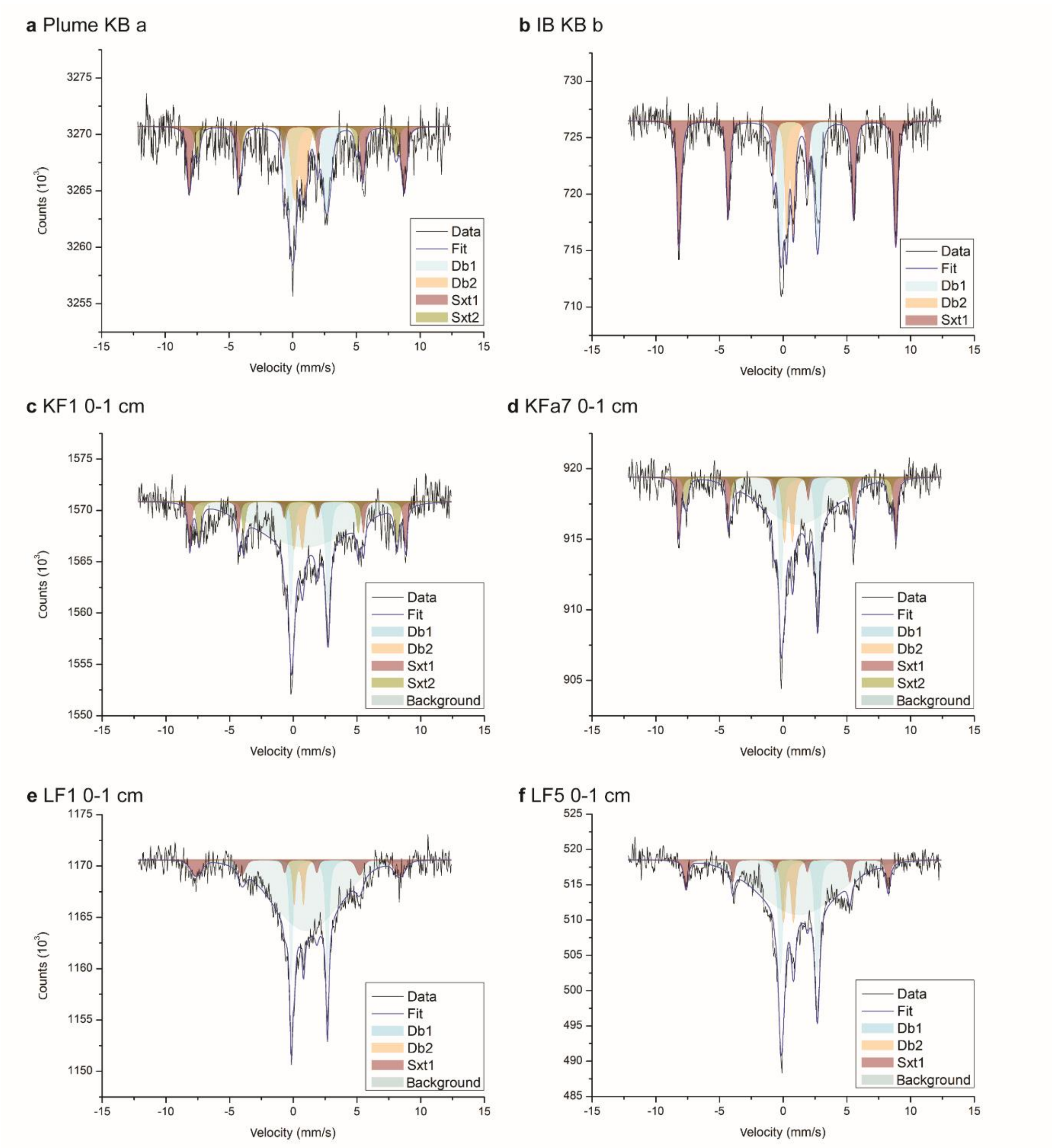

Supplementary Figure 9: Mössbauer spectra collected at 5 K. All samples showed a wide doublet (Db1; light blue) that can be attributed to the presence of a high-spin Fe(II) mineral phase in all samples. Samples a)-d) showed two additional sextet features (Sxt1 and Syt2; red and olive) that indicate the presence of a magnetically-ordered mineral phase at 77K. The wide hyperfine field (>50 T) suggests hematite as iron(III) oxide likely to be present in these samples. The narrow sextet (Sxt2; olive) can most likely be attributed to a poorly-crystalline Fe(III) mineral phase, similar to ferrihydrite, that shows magnetic ordering at 5 K.

Supplementary Tables

Supplementary Table 1: Results from iron extractions of glacial source material. For identity of the samples see Supplementary Table 6. Ascorbate extractions = time-course extractions with bicarbonate-buffered ascorbate. microbial extractions =time-course extractions with a culture of *Shewanella frigidimarina*. $M_{(0)}$ = amount of ascorbate/microbially extractable iron; v/a = apparent rate constant, reducibility of FeA or FeM; initial rate = initial reduction rate as a measure of lability of FeA or FeM; 1+1/v= heterogeneity parameter, indicating the complexitiy of the composition of the extracted minerals (see Material and Method for detailed description of the parameters). HCl-extractions= sequential extractions with different concentrations of HCl.

	ascorbate extractions					microbial extractions					HCl extractions (in µmol gdw^{-1})				
Sample	$M_{(0)}$ (µmol g dw^{-1})	v/a * 10 000 (s^{-1})	Initial rate * 100 (µmol g dw^{-1} s^{-1})	1+1/v	R^2	$M_{(0)}$ (µmol g dw^{-1})	v/a * 10 000 (s^{-1})	Initial rate * 100 (µmol g dw^{-1} s^{-1})	1+1/v	R^2	Fe(II) 0.5 M	Fe(III) 0.5 M	Fe(II) 6 M	Fe(III) 6 M	Total Fe
IB KB a	4.54	2.47	0.112	2.36	0.98	Nd	Nd	Nd	Nd	Nd	3.0	6.7	140	320	470
IB KB b	2.62	4.33	0.113	2.29	1.00	4.78	0.0156	0.00103	1.02	0.99	3.1	4.7	110	330	450
IB KB c	5.93	0.608	0.0360	1.19	1.00	10.7	0.0143	0.00243	0.943	0.99	7.9	4.5	350	220	580
IB KB d	8.36	0.565	0.0472	1.00	1.00	26.2	0.103	0.0269	2.457	0.99	8.8	6.1	100	670	780
IB KB e	6.55	2.82	0.184	2.18	0.99	12.9	0.324	0.0417	4.25	0.96	5.5	5.4	190	460	660
IB KB f	7.18	0.460	0.0330	1.10	1.00	24.9	0.0105	0.00261	1.01	1.0	13.	2.6	100	540	660
IB KV	21.3	1.21	0.257	1.48	1.00	50.8	0.0307	0.01563	1.88	0.95	56	20	220	170	470
Plume KB a	28.5	2.49	0.710	2.02	0.99	Nd	Nd	Nd	Nd	Nd	59.	34	230	190	510
Plume KB b	30.7	4.26	1.31	2.59	0.98	Nd	Nd	Nd	Nd	Nd	65	29	130	97	320
Plume KB c	26.8	2.70	0.724	1.57	1.00	77.6	0.0482	0.0374	2.06	1.0	49	18	280	370	720
Plume KV	37.4	2.06	0.769	1.12	0.99	146	0.0431	0.0629	1.88	0.99	62	32	200	200	490
ALB a	29.7	0.355	0.111	0.932	1.00	82.6	0.0355	0.0293	1.57	1.0	65	14	530	240	850
ALB b	52.9	0.450	0.242	1.02	1.00	Nd	Nd	Nd	Nd	Nd	110	510	630	170	1400
MLB a	23.4	0.443	0.104	1.08	1.00	Nd	Nd	Nd	Nd	Nd	46	15	61	360	490
MLB b	20.9	0.658	0.143	1.00	0.99	95.2	0.0337	0.0321	2.17	0.99	39	7.9	96	380	530
FB	24.5	0.413	0.102	1.08	1.00	Nd	Nd	Nd	Nd	Nd	43	7.6	51	410	510
BAY	16.9	0.568	0.101	0.882	1.00	176	0.0152	0.0269	1.11	1.0	52	12	280	550	890
River DF	5.95	0.578	0.0343	1.31	1.00	17.5	0.0434	0.00761	1.96	0.99	3.2	4.9	270	540	820

Nd= not determined

Supplementary Table 2 – Overview on Mössbauer spectra fitting parameters. Temp. – temperature during measurement; Phase – fitted compound, Db: doublet, Sxt: Sextett, center shift (CS in mm/s); quadrupole splitting (ΔE_Q in mm/s); quadrupole shift (ε in mm/s); hyperfine field (B_{hf} in T), R.A. – relative abundance (in %), χ^2 as goodness of fit and identified (iron) mineral phase: Fe(II), Fe(III), Hematite (Hem), Ferrihydrite (Fh), unknown (?)

**Sample**	**Temp.**	**Phase**	**CS**	**ΔE_Q**	**ε**	**B_{hf}**	**R.A.**	**χ^2**	**mineral**
	**K**		**mm/s**	**mm/s (±)**	**mm/s**	**T (±)**	**% (±)**		**phase**
Plume KB a	77	Db1	1.23	2.84 (0.07)			58.9 (0.3)	0.62	Fe(II)
		Db2	0.46	0.69 (0.09)			23.3 (0.7)		Fe(III)
		Sxt	0.45		-0.15	52.3 (0.29)	17.8 (1.6)		Hem
	5	Db1	1.23	2.88 (0.25)			31.6 (2.9)	0.75	Fe(II)
		Db2	0.52	0.78 (0.13)			20.6 (1.7)		?
		Sxt1	0.48		-0.15	52.3 (0.17)	33.1 (4.6)		Hem
		Sxt2	0.41		-0.09	48.2 (0.11)	14.7 (5.2)		Fh
IB KB b	77	Db1	1.22	2.83 (0.08)			28.1 (0.6)	0.69	Fe(II)
		Db2	0.42	0.68 (0.10)			30.6 (0.2)		Fe(III)
		Sxt	0.46		-0.13	52.7 (0.14)	41.3 (1.9)		Hem
	5	Db1	1.26	2.88 (0.14)			30.3 (2.0)	0.95	Fe(II)
		Db2	0.54	0.53 (0.20)			17.3 (1.3)		?
		Sxt1	0.46		-0.14	52.8 (0.17)	40.6 (4.3)		Hem
		Sxt2	0.36		-0.17	49.7 (0.13)	11.8 (5.1)		Fh
KF1 0-1 cm	77	Db1	1.24	2.78 (0.18)			63.2 (0.7)	0.69	Fe(II)
		Db2	0.55	0.52 (0.19)			15.7 (0.8)		Fe(III)
		Sxt	0.48		-0.18	52.5 (0.21)	21.1 (1.3)		Hem
	5	Db1	1.26	2.88 (0.23)			17.7 (2.8)	0.62	Fe(II)
		Db2	0.42	0.64 (0.29)			9.5 (3.4)		?
			background – paramagnetic relaxation				46.6 (-)		-
		Sxt1	0.48		-0.14	52.8 (0.31)	17.1 (5.2)		Hem
		Sxt2	0.50		-0.12	49.6 (0.21)	9.1 (3.9)		Fh
KFa7 0-1 cm	77	Db1	1.24	2.81 (0.06)			48.7 (0.3)	0.71	Fe(II)
		Db2	0.47	0.75 (0.09)			41.0 (1.1)		Fe(III)
		Sxt	0.46		-0.20	50.9 (0.13)	10.3 (2.1)		Hem
	5	Db1	1.27	2.90 (0.16)			15.5 (3.8)	0.89	Fe(II)
		Db2	0.39	0.66 (0.13)			6.8 (1.4)		?
			background – paramagnetic relaxation				54.3 (-)		
		Sxt1	0.48		-0.13	52.5 (0.21)	11.0 (3.6)		Hem
		Sxt2	0.50		-0.12	48.2 (0.17)	12.2 (2.9)		Fh
LF1 0-1 cm	77	Db1	1.24	2.78 (0.06)			80.4 (0.7)	0.60	Fe(II)
		Db2	0.52	0.73 (0.09)			19.6 (1.1)		Fe(III)
	5	Db1	1.26	2.85 (0.19)			15.3 (4.3)	0.63	Fe(II)

		Db2	0.44	0.72 (0.11)			5.5 (1.9)		?
			background – paramagnetic relaxation				68.2		-
		Sxt1	0.48		-0.11	49.1 (0.23)	11.0 (3.9)		Fh
LF5 0-1 cm	77	Db1	1.24	2.78 (0.09)			72.5 (0.4)	0.74	Fe(II)
		Db2	0.49	0.80 (0.10)			27.5 (0.6)		Fe(III)
	5	Db1	1.24	2.88 (0.13)			19.0 (1.9)	0.94	Fe(II)
		Db2	0.42	0.78 (0.19)			9.9 (2.3)		?
			background – paramagnetic relaxation				61.6 (-)		-
		Sxt1	0.48		-0.16	49.2 (0.24)	9.5 (6.1)		Fh

Supplementary Table 3: Statistical analysis of the increases of the amount and lability of FeA over distance at the sediment surface. The LF_{short} transect only contains station LF1-LF5.

Transect-parameter	R^2 of linear regression	p-value (significance)
KFa- amount of FeA)	0.96	9.7 x 10^{-5} (***)
KFb-- amount of FeA)	0.92	0.0090 (**)
LF-- amount of FeA))	0.62	0.012 (*)
LF_{short}-- amount of FeA)	0.95	0.0051 (**)
DF-- amount of FeA))	0.90	0.013 (*)
KFa-lability of FeA	0.97	3.6 x 10^{-5} (***)
KFb- lability of FeA	0.92	0.0095 (**)
LF- lability of FeA	0.82	0.39 (not significant)
LF_{short}- lability of FeA	0.93	0.0075 (**)
DF- lability of FeA	0.91	0.013 (*)

Supplementary Table 4: Results from sequential HCl and ascorbate endpoint extractions of sediment in the DF transect. See Supplementary Table 6 for information about station locations. Numbers behind station labels indicate sediment depth in cm.

Sample	Fe(II) 0.5 M HCl (µmol g dw^{-1})	Fe(III) 0.5 M HCl (µmol g dw^{-1})	Fe(II) 6 M HCl (µmol g dw^{-1})	Fe(III) 6 M HCl (µmol g dw^{-1})	Mn 0.5 M HCl (µmol g dw^{-1})	Mn 6 M HCl (µmol g dw^{-1})	Mn Ascorbate (µmol g dw^{-1})
DF1 0-1	3.2	3.8	220	370	25	3.6	9.0
DF1 1-2	3.9	4.1	230	400	26	2.8	4.4
DF1 2-3	2.9	7.8	230	400	31	3.0	Nd
DF1 3-4	3.6	5.3	230	380	35	3.6	7.1
DF1 4-6	9.3	0.5	180	260	22	2.3	Nd
DF1 6-8	10	1.6	210	340	21	2.8	2.4
DF1 8-10	10	1.7	230	380	22	2.9	Nd
DF1 10-13	10	1.9	250	390	23	3.2	4.2
DF2 0-1	0.6	12	220	350	27	2.6	8.0
DF3 0-1	0.9	24	240	370	37	6.7	30
DF3 1-2	0.7	23	230	410	66	16	56
DF3 2-3	0.5	21	200	420	110	21	Nd
DF3 3-4	0.7	46	230	420	100	8.6	220
DF3 4-6	30	1.8	200	340	37	2.6	Nd
DF3 6-8	28	0.9	190	290	59	3.4	11
DF3 8-10	48	0.3	230	370	81	17	Nd
DF3 10-13	42	0.0	140	210	42	1.6	9.7
DF4 0-1	0.8	30	140	240	40	7.8	32
DF5 0-1	0.9	33	170	270	46	11	57
DF5 1-2	0.8	29	130	360	60	30	33
DF5 2-3	0.6	29	130	390	98	31	Nd
DF5 3-4	1.0	38	180	310	130	27	30
DF5 4-6	1.2	67	170	320	100	8.2	Nd
DF5 6-8	59	36	190	320	58	2.4	16
DF5 8-10	46	0.3	160	260	47	1.8	Nd
DF5 10-13	48	0.7	180	160	49	1.4	12

Supplementary Table 5: Station names and coordinates. Main stations, where all analyses were done in depth-resolution are highlighted in bold.

Station/ source names	RIS project name	Coordinates	Sampling date (month/year)	Water depth (m)	Distance from glacial source (m)	Remarks
**KF1**	JE	78° 53.223 N<br>12° 34.287 E	06/2017	57	688	Data from Fe extractions, sulfate reduction rates and pore water Fe and Mn data already published[15]
KFa2	JK	78° 53.489 N<br>12° 24.652 E	06/2017	83	4172	
**KFa3**	IA	78° 53.750 N<br>12° 20.220 E	06/2017	54	5815	
KFa4	JJ	78° 54.488 N<br>12° 17.377 E	06/2017	55	7176	
**KFa5**	F	78° 55.096 N<br>12° 15.929 E	06/2017	105	8112	Data from Fe extractions, sulfate reduction rates and pore water Fe and Mn data already published[15]
KFa6	JI	78° 56.350 N<br>11° 08.057 E	06/2017	193	11542	
**KFa7**	T	78° 58.060 N<br>11° 52.910 E	06/2017	336	18509	Data from Fe extractions, sulfate reduction rates and pore water Fe and Mn data already published[15]
KFb2	JD	78° 54.724 N<br>12° 25.113 E	06/2017	73	4953	
**KFb3**	JC	78° 55.769 N<br>12° 23.437 E	06/2017	53	6711	
KFb4	JB	78° 57.231 N<br>12° 18.693 E	06/2017	68	9916	
**KFb5**	P	78° 57.990 N<br>12° 15.361 E	06/2017	92	11756	Sulfate reduction rates already published[16]
**LF1**	JL	79° 20.031 N<br>11° 42.729 E	07/2017	66	3224	
LF2	JM	79° 18.569 N<br>11° 35.806 E	07/2017	139	5099	
LF3	JN	79° 17.172 N<br>11° 36.726 E	07/2017	221	7715	
LF4	JO	79° 15.895 N<br>11° 38.746 E	07/2017	252	10130	
**LF5**	JP	79° 14.812 N<br>11° 39.760 E	07/2017	260	12170	
LF6	JQ	79° 13.464 N<br>11° 41.626 E	07/2017	266	14708	
LF7	JR	79° 11.438 N<br>11° 46.148 E	07/2017	369	18733	
**LF8**	CN	79° 08.999 N<br>11° 44.230 E	06/2017	350	23176	
LF9	JS	79° 07.060 N<br>11° 39.908 E	07/2017	310	26611	
**DF1**	KB	78° 48.060 N<br>15° 19.390 E	08/2018	42	2506	Sulfate reduction rates already published[16]
DF2	KC	78° 46.380 N<br>15° 18.320 E	08/2018	58	5585	
**DF3**	KD	78° 43.960 N<br>15° 17.560 E	08/2018	89	10021	Sulfate reduction rates already published[16]
DF4	KE	78° 41.410 N<br>15° 17.990 E	08/2018	87	14696	
**DF5**	C	78° 39.190 N<br>15° 19.150 E	08/2018	106	18760	Sulfate reduction rates already published[16]

Supplementary Table 6: Overview of glacial sources sampled.

Sample name	Type of sample	Sampling location	Sampling date (month/year)	Remarks
IB KB a	Iceberg, sampled in front of Kronebreen in KF	Close to station KF1	06/2017	
IB KB b	Iceberg, sampled in front of Kronebreen in KF	Close to station KF1	06/2017	
IB KB c	Iceberg, sampled in front of Kronebreen in KF	Close to station KF1	06/2017	
IB KB d	Iceberg, sampled in front of Kronebreen in KF	Stranded in front of Ny-Alesund	04/2018	
IB KB e	Iceberg, sampled in front of Kronebreen in KF	Close to station KF1	07/2018	
IB KB f	Iceberg, sampled in front of Kronebreen in KF	Close to Plume KB c	07/2018	
IB KV	Iceberg, sampled in front of Kongsvegen in KF	Close to Plume KV	07/2018	
Plume KB a	Proglacial plume sampled in front of Kronebreen KF	78° 52.085 N<br>12° 32.835 E	06/2017	
Plume KB b	Proglacial plume sampled in front of Kronebreen KF	78° 52.753 N<br>12° 33.372 E	06/2017	
Plume KB c	Proglacial plume sampled in front of Kronebreen KF	78° 53.175 N<br>12° 34.884 E	07/2018	
Plume KV	Proglacial plume sampled in front of Kongsvegen KF	78° 51.640 N<br>12° 34.009 E	07/2018	
ALB a	Meltwater stream from Austre Lovenbreen, KF	78° 54.248 N<br>12° 09.671 E	06/2017	
ALB b	Meltwater stream from Austre Lovenbreen, KF	78° 54.248 N<br>12° 09.671 E	07/2018	
MLB a	Meltwater stream from Midre Lovenbreen, KF	78° 53.614 N<br>12° 04.245 E	06/2017	
MLB b	Meltwater stream from Midre Lovenbreen, KF	78° 53.614 N<br>12° 04.245 E	07/2018	
FB	Meltwater stream from Ferringbreen, KF	78° 59.543 N<br>12° 24.896 E	06/2017	
BAY	Bayelva river, KF	78° 56.007 N<br>11° 51.952 E	07/2018	
River DF	Main glacial stream entering DF at its head	78° 48.84 N<br>15° 22.26 E	08/2018	

Supplementary Table 7: Results of TOC and C:N quantification in sediment from the transects. See Supplementary Table 8 for information about station locations. Numbers behind station labels indicate sediment depth.

Sample	C:N	TOC (% of dw)
KF1 0-1	33.3	1.31
KF1 1-2	35.4	1.51
KF1 2-3	48.7	1.25
KF1 3-4	54.6	0.95
KF1 4-6	69.7	0.91
KF1 6-8	61.9	0.87
KF1 8-10	69.1	0.80
KF1 10-13	66.3	0.87
KFa2 0-2	45.7	1.63
KFa3 0-1	27.2	1.24
KFa3 1-2	38.3	1.27
KFa3 2-3	40.6	1.22
KFa3 3-4	46.2	1.28
KFa3 4-6	39.1	1.06
KFa3 6-8	27.7	1.28
KFa3 8-10	33.7	1.44
KFa3 10-13	32.8	1.30
KFa4 0-2	31.5	1.42
KFa5 0-1	18.2	1.88
KFa5 1-2	28.0	1.41
KFa5 2-3	27.8	1.41
KFa5 3-4	26.1	1.43
KFa5 4-6	26.9	1.37
KFa5 6-8	27.1	1.47
KFa5 8-10	32.1	1.54
KFa5 10-13	35.4	1.50
KFa6 0-2	19.4	1.80
KFa7 0-1	39.4	3.97
KFa7 1-2	16.0	2.32
KFa7 2-3	16.2	2.21
KFa7 3-4	16.9	2.31
KFa7 4-6	16.1	2.22
KFa7 6-8	17.5	2.09
KFa7 8-10	18.8	2.00
KFa7 10-13	18.6	1.96
KFb2 0-2	37.3	1.39
KFb3 0-1	51.1	2.03
KFb3 1-2	33.2	1.28
KFb3 2-3	41.3	1.28
KFb3 3-4	34.4	1.13
KFb3 4-6	37.0	1.09
KFb3 6-8	38.6	1.30
KFb3 8-10	45.3	1.36
KFb3 10-13	38.2	1.40
KFb4 0-2	33.9	1.45
KFb5 0-1	60.3	2.96
KFb5 1-2	35.1	2.17
KFb5 2-3	30.9	1.44
KFb5 3-4	32.4	1.51
KFb5 4-6	30.3	1.23
KFb5 6-8	34.4	1.49
KFb5 8-10	37.3	1.67
KFb5 10-13	40.1	1.57
LF1 0-1	42.8	0.97

LF1 1-2	56.7	0.83
LF1 2-3	52.9	0.83
LF1 3-4	48.5	0.84
LF1 4-6	40.7	0.61
LF1 6-8	42.6	0.88
LF1 8-10	48.9	0.77
LF1 10-13	43.6	0.76
LF2 0-2	48.4	1.20
LF3 0-2	27.7	1.53
LF4 0-2	34.5	1.26
LF5 0-1	34.6	1.84
LF5 1-2	32.5	1.47
LF5 2-3	28.7	1.53
LF5 3-4	26.1	1.36
LF5 4-6	27.8	1.37
LF5 6-8	24.3	0.99
LF5 8-10	29.5	1.17
LF5 10-13	26.0	1.15
LF6 0-2	34.8	1.45
LF7 0-2	24.2	1.77
LF8 0-1	49.5	3.53
LF8 1-2	44.9	3.40
LF8 2-3	44.9	3.40
LF8 3-4	23.1	2.07
LF8 4-6	24.6	2.43
LF8 6-8	nd	nd
LF8 8-10	21.3	2.15
LF8 10-13	26.0	1.86
LF9 0-2	21.7	1.85
DF1 0-1	38.4	1.03
DF1 1-2	14.1	0.46
DF1 2-3	35.6	1.05
DF1 3-4	47.2	1.25
DF1 4-6	37.6	0.76
DF1 6-8	46.7	1.05
DF1 8-10	36.9	0.83
DF1 10-13	41.0	0.98
DF2 0-1	34.7	1.28
DF3 0-1	25.5	1.55
DF3 1-2	27.9	1.44
DF3 2-3	33.4	1.65
DF3 3-4	29.1	1.45
DF3 4-6	30.3	1.34
DF3 6-8	26.2	1.20
DF3 8-10	29.6	1.34
DF3 10-13	26.4	1.18
DF4 0-1	24.2	1.56
DF5 0-1	20.5	1.67
DF5 1-2	23.2	1.74
DF5 2-3	23.7	1.68
DF5 3-4	24.9	1.58
DF5 4-6	25.7	1.59
DF5 6-8	24.8	1.49
DF5 8-10	26.4	1.68
DF5 10-13	29.1	1.65

Supplementary Table 8: Results of ascorbate time-course extractions of sediment in transects. See Supplementary Table 5 for information about station locations. Numbers behind station labels indicate sediment depth. $M_{(0)}$ = amount of ascorbate extractable iron; v/a = reducibility; initial rate = lability; 1+1/v= composition (see Material and Method for detailed description of the parameters).

Sample	$M_{(0)}$ (µmol g dw^{-1})	v/a * 10 000 (s^{-1})	Initial rate * 100 (µmol g dw^{-1} s^{-1})	1+1/v	R^2
KF1 0-1	19.3	2.27	0.44	1.85	0.99
KF1 1-2	17.8	6.22	1.11	2.80	0.99
KF1 3-4	17.9	2.14	0.38	2.12	0.99
KF1 6-8	13.5	1.90	0.26	1.91	0.99
KF1 10-13	9.69	1.61	0.16	1.74	1.0
KFa2 0-2	27.5	2.47	0.68	2.01	1.0
KFa3 0-1	25.6	4.20	1.07	2.36	0.99
KFa3 1-2	31.4	1.91	0.60	1.67	1.0
KFa3 3-4	18.3	6.67	1.22	3.02	0.98
KFa3 6-8	22.9	3.03	0.69	2.56	0.98
KFa3 10-13	21.0	4.27	0.90	2.28	0.99
KFa4 0-2	33.0	2.81	0.93	2.18	0.99
KFa5 0-1	50.6	3.51	1.78	1.83	1.0
KFa5 1-2	48.7	3.06	1.49	1.96	0.99
KFa5 3-4	37.8	4.48	1.69	2.35	0.99
KFa5 6-8	35.6	1.07	0.38	1.81	0.99
KFa5 10-13	15.7	2.85	0.45	1.86	1.0
KFa6 0-2	66.3	3.73	2.47	1.88	1.0
KFa7 0-1	176	4.81	8.45	1.20	0.99
KFa7 1-2	136	5.01	6.81	1.66	0.99
KFa7 3-4	79.9	1.32	1.05	1.76	0.99
KFa7 6-8	63.5	1.01	0.64	1.31	1.0
KFa7 10-13	57.4	2.65	1.52	2.14	0.99
KFb2 0-2	25.9	2.59	0.67	1.96	0.99
KFb3 0-1	36.0	2.01	0.72	2.06	0.99
KFb3 1-2	27.9	2.74	0.76	2.38	0.99
KFb3 3-4	26.7	3.85	1.03	2.69	0.98
KFb3 6-8	26.9	2.39	0.64	2.31	0.97
KFb3 10-13	24.9	1.87	0.46	1.90	0.99
KFb4 0-2	41.6	2.47	1.03	1.70	0.99
KFb5 0-1	71.6	2.56	1.83	1.20	0.99
KFb5 1-2	41.1	2.75	1.13	1.40	1.0
KFb5 3-4	38.0	1.84	0.70	1.44	1.0
KFb5 6-8	32.4	1.41	0.46	1.67	1.0
KFb5 10-13	32.0	0.757	0.24	1.34	1.0
LF1 0-1	30.8	2.61	0.80	1.77	0.99
LF1 1-2	28.1	1.51	0.42	1.78	0.99
LF1 3-4	26.3	2.22	0.58	2.22	0.97
LF1 6-8	19.7	0.832	0.16	1.67	0.99
LF1 10-13	17.8	0.439	0.08	1.10	0.99
LF2 0-2	43.6	2.32	1.01	2.03	0.99
LF3 0-2	50.6	3.34	1.69	2.30	0.99
LF4 0-2	57.4	3.52	2.02	2.20	1.00
LF5 0-1	72.3	2.95	2.13	1.03	1.0
LF5 1-2	64.7	4.83	3.12	1.47	1.0
LF5 3-4	49.0	3.01	1.47	2.12	0.99
LF5 6-8	41.5	3.51	1.45	1.57	0.99
LF5 10-13	33.8	1.32	0.45	1.66	1.00
LF6 0-2	48.4	4.53	2.19	2.45	0.99
LF7 0-2	52.4	1.91	1.00	2.00	0.98
LF8 0-1	89.0	3.83	3.41	1.06	1.00

LF8 1-2	65.3	3.21	2.10	2.41	0.99
LF8 3-4	55.4	1.49	0.82	1.83	1.0
LF8 6-8	66.9	2.58	1.72	2.42	0.98
LF8 10-13	49.9	0.921	0.46	1.75	0.99
LF9 0-2 cm	75.2	1.49	1.12	1.93	0.99
DF1 0-1	5.67	5.90	0.33	2.40	0.99
DF1 1-2	4.54	1.20	0.05	1.95	0.97
DF1 3-4	5.27	3.50	0.18	2.33	0.98
DF1 6-8	4.85	0.797	0.04	2.06	0.93
DF1 10-13	4.53	4.02	0.18	2.43	0.97
DF2 0-1	8.08	11.2	0.91	2.78	0.98
DF3 0-1	25.3	14.1	3.57	3.06	0.97
DF3 1-2	21.9	11.7	2.56	3.16	0.95
DF3 3-4	38.3	10.0	3.84	1.47	0.99
DF3 6-8	18.5	1.89	0.35	2.40	0.99
DF3 10-13	19.0	0.746	0.14	1.83	0.97
DF4 0-1	29.1	11.3	3.30	2.34	0.97
DF5 0-1	38.9	23.1	8.99	3.18	0.93
DF5 1-2	30.5	21.6	6.60	3.10	0.96
DF5 3-4	35.2	8.80	3.10	2.61	1.0
DF5 6-8	70.7	12.8	9.05	2.58	0.95
DF5 10-13	33.8	0.994	0.34	1.73	0.99

Supplementary Table 9: Results of MFeR extractions of sediment in transects. See Supplementary Table 5 for information about station locations. Numbers behind station labels indicate sediment depth. . $M_{(0)}$ = amount of ascorbate extractable iron; v/a = reducibility; initial rate = lability; 1+1/v= composition (see Material and Method for detailed description of the parameters).

Sample	$M_{(0)}$ (µmol g dw^{-1})	v/a * 10 000 (s^{-1})	Initial rate * 100 (µmol g dw^{-1} s^{-1})	1+1/v	R^2
KF1 0-1	25.6	0.382	0.101	2.31	0.95
KF1 10-13	12.3	0.131	0.0221	1.42	0.96
KFa5 0-1	58.4	0.484	0.281	2.40	0.97
KFa5 10-13	24.8	0.191	0.0591	1.78	0.96
KFa7 0-1	300	1.52	4.55	1.97	1.00
KFa7 10-13	48.9	0.301	0.151	1.71	0.99
KFb3 0-1	44.3	0.25	0.113	1.66	0.99
KFb3 10-13	27.5	0.172	0.0574	1.34	0.99
KFb5 0-1	104	0.773	0.801	1.77	0.99
KFb5 10-13	34.2	0.122	0.0462	1.36	0.96
LF1 0-1	46.0	0.301	0.141	1.96	0.97
LF1 10-13	11.6	0.111	0.0184	1.33	0.94
LF3 0-2	48.1	0.792	0.387	2.41	0.98
LF5 0-1	97.3	0.522	0.519	1.69	0.97
LF5 10-13	38.0	0.113	0.0413	1.26	0.96
DF1 0-1	11.5	0.0331	0.00473	0.954	0.99
DF1 10-13	12.1	0.0704	0.00839	1.83	0.95
DF2 0-1	20.8	0.0591	0.0126	1.54	0.99
DF3 0-1	48.9	0.242	0.116	2.28	0.97
DF3 10-13	29.1	0.0439	0.0134	1.72	0.98
DF4 0-1	59.7	0.292	0.175	3.09	0.95
DF5 0-1	72.5	0.343	0.249	2.64	0.97
DF5 6-8	70.8	0.504	0.350	3.47	0.93
DF5 6-8	126	0.626	0.802	3.93	0.93
DF5 10-13	42.8	0.0211	0.00917	0.963	0.98

Supplementary References

1. Elverhøi, A., Lønne, Ø. & Seland, R. Glaciomarine sedimentation in a modern fjord environment, Spitsbergen. *Polar Res.* **1**, 127–150 (1983).
2. Howe, J. A., Moreton, S. G., Morri, C. & Morris, P. Multibeam bathymetry and the depositional environments of Kongsfjorden and Krossfjorden, western Spitsbergen, Svalbard. *Polar Res.* **22**, 301–316 (2003).
3. Løvlie, R., Torsvik, T., Jelenska, M. & Levandowski, M. Evidence for detrital remanent magnetization carried by hematite in Devonian red beds from Spitsbergen; palaeomagnetic implications. *Geophys. J. R. Astron. Soc.* **79**, 573–588 (1984).
4. Jeleńska, M. & Lewandowski, M. A palaeomagnetic study of Devonian sandstone from Central Spitsbergen. *Geophys. J. R. Astron. Soc.* **87**, 617–632 (1986).
5. Dallmann, W. K. *Geoscience Atlas of Svalbard*. (Norsk polarinstitutt, 2015).
6. Piepjohn, K. & Dallmann, W. K. Stratigraphy of the uppermost Old Red Sandstone of Svalbard (Mimerdalen Subgroup). *Polar Res.* **33**, 19998 (2014).
7. Meslard, F., Bourrin, F., Many, G. & Kerhervé, P. Suspended particle dynamics and fluxes in an Arctic fjord (Kongsfjorden, Svalbard). *Estuar. Coast. Shelf Sci.* **204**, 212–224 (2018).
8. Lydersen, C., Kovacs, K. M. & Lydersen, E. Rust-colored bearded (Erignathus barbatus) and ringed (Phoca hispida) seals from Svalbard, Norway. *J. Mammal.* **82**, 225–230 (2001).
9. Elverhøi, A., Liestøl, O. & Nagy, J. *Glacial erosion, sedimentation and microfauna in the inner part of Kongsfjorden, Spitsbergen*. (Norsk Polarinstitutt Skrifter, 1980).
10. Svendsen, H. *et al.* The physical environment of Kongsfjorden – Krossfjorden, an Arctic fjord system in Svalbard. *Polar Res.* **21**, 133–166 (2002).
11. Kvam, M. H. Deposits and processes on the tide-influenced fjord- head delta in Dicksonfjorden , Svalbard. *Masters Thesis, UiT The Arctic University of Norway* (2018).
12. Murad, E. & Schwertmann, U. The Mössbauer spectrum of ferrihydrite and its relations to those of other iron oxides. *Am. Mineral.* **65**, 1044–1049 (1980).
13. Lydersen, C. *et al.* The importance of tidewater glaciers for marine mammals and seabirds in Svalbard, Norway. *J. Mar. Syst.* **129**, 452–471 (2014).
14. Węsławski, J. M. *et al.* Can seabirds modify carbon burial in fjords? *Oceanologia* **59**, 603–611 (2017).
15. Laufer, K., Michaud, A., Røy, H. & Jørgensen, B. Reactivity of iron minerals in the seabed towards microbial reduction – a comparison of different extraction techniques. *Geomicrobiol. J.* **37**, 170–189 (2020).
16. Jørgensen, B. B., Laufer, K., Michaud, A. B. & Wehrmann, L. M. Biogeochemistry and microbiology of high Arctic marine sediment ecosystems- case study Svalbard fjords. *Limnol. Oceanogr.* **20**, 1–20 (2020).
